# Occurrence, Geochemistry and Speciation of Elevated Arsenic Concentrations in a Fractured Bedrock Aquifer System

**DOI:** 10.1007/s00244-021-00887-3

**Published:** 2021-09-14

**Authors:** Ellen McGrory, Tiernan Henry, Peter Conroy, Liam Morrison

**Affiliations:** Earth and Ocean Sciences, School of Natural Sciences and Ryan Institute, Environmental, Marine and Energy Research, National University of Ireland, University Road, Galway, H91 TK33 Ireland

## Abstract

The presence of elevated arsenic concentrations (≥ 10 µg L^−1^) in groundwaters has been widely reported in areas of South-East Asia with recent studies showing its detection in fractured bedrock aquifers is occurring mainly in regions of north-eastern USA. However, data within Europe remain limited; therefore, the objective of this work was to understand the geochemical mobilisation mechanism of arsenic in this geologic setting using a study site in Ireland as a case study. Physicochemical (pH, Eh, d-O_2_), trace metals, major ion and arsenic speciation samples were collected and analysed using a variety of field and laboratory-based techniques and evaluated using statistical analysis. Groundwaters containing elevated dissolved arsenic concentrations (up to 73.95 µg L^−1^) were characterised as oxic-alkali groundwaters with the co-occurrence of other oxyanions (including Mo, Se, Sb and U), low dissolved concentrations of Fe and Mn, and low Na/Ca ratios indicated that arsenic was mobilised through alkali desorption of Fe oxyhydroxides. Arsenic speciation using a solid-phase extraction methodology (*n* = 20) showed that the dominant species of arsenic was arsenate, with pH being a major controlling factor. The expected source of arsenic is sulphide minerals within fractures of the bedrock aquifer with transportation of arsenic and other oxyanion forming elements facilitated by secondary Fe mineral phases. However, the presence of methylarsenical compounds detected in groundwaters illustrates that microbially mediated mobilisation processes may also be (co)-occurring. This study gives insight into the geochemistry of arsenic mobilisation that can be used to further guide research needs in this area for the protection of groundwater resources.

**Supplementary Information:**

The online version contains supplementary material available at 10.1007/s00244-021-00887-3.

The presence of geogenic arsenic in groundwater remains a major health concern affecting approximately 226 million people in the world (Garelick et al. 2009; Smedley and Kinniburgh 2013; Murcott 2012). In many regions, groundwater remains an important source of drinking water which is a critical exposure pathway for arsenic (Smedley and Kinniburgh 2013). For example, an estimated 2 million people are exposed to drinking water containing elevated arsenic concentrations sourced from domestic wells in the USA (Ayotte et al. 2017).

The International Agency for Research on Cancer (IARC) classifies arsenic as a Group I carcinogen (IARC 2012). Short-term or acute exposure to high arsenic concentrations can lead to the development of arsenicosis (van Halem et al. 2009; Naujokas et al. 2013). In contrast, it has been demonstrated that long term or chronic exposure to arsenic concentrations (< 100 μg L^−1^) can also lead to similar adverse health effects (Moon et al. 2012; Bräuner et al. 2014; Stea et al. 2014; Tsuji et al. 2014; He et al. 2020, 2021; Wei et al. 2021). This is an important consideration as many people rely on private wells for drinking water. Large proportions of rural communities globally report arsenic concentrations in the range of 10–100 µg L^−1^ due to the dispersed occurrence of arsenic in the environment and the frequent reliance on private wells as drinking water sources (Ryan et al. 2013; Bondu et al. 2017; Zheng et al. 2017).

The pentavalent form of arsenic, arsenate (As^V^) and the trivalent form, arsenite (As^III^), are the most detected forms in groundwater (Zecchin et al. 2021). Arsenate forms the oxyanions H_2_AsO_4_^−^ and HAsO_4_^−^ in oxidising environments in natural waters, while arsenite forms the oxyanions H_3_AsO_3_^0^ and H_2_AsO_3_^−^ in reducing environments with the degree of protonation depending on pH (Garelick et al. 2009; Herath et al. 2016). Organic arsenic species may be present in groundwater because of biological activity, but their relative concentrations may be negligible (Cullen and Reimer 1989; Smedley and Kinniburgh 2013; Schreiber 2021; Stetson et al. 2021). These methylated organic forms of arsenic include the pentavalent dimethylarsinic acid (DMA^V^) and methylarsonic acid (MA^V^) which are stable mammalian metabolites (Lord et al. 2012).

The mobility, toxicity, adsorption and biogeochemical cycling of arsenic depends on the oxidation state, or speciation of arsenic (Garelick 2009; Schreiber, 2021). While the geology, hydrogeology and geochemistry of the aquifer system remain important controls on the mobility of trace elements within the solid-aqueous environment (Garelick et al. 2009; Wei et al. 2021), the redox parameters pH and Eh are the dominant geochemical factors controlling oxyanion forming elements in natural waters (Smedley and Kinniburgh 2013; Herath et al. 2016). Two geochemical triggers are responsible for arsenic mobilisation: one to release arsenic from the host rock and another to retain arsenic in the groundwater after the initial release (Smedley and Kinniburgh 2013). Other trace elements present in the host rock or minerals may also be mobilised by the same mechanism as arsenic and thus may also be elevated, e.g. U and Mo.

In Asia, groundwaters with elevated arsenic are generally associated with unconsolidated Quaternary alluvial sediments, with geochemical and hydrogeological conditions favouring the mobilisation of arsenic (Ryan et al. 2011; Blake and Peters 2015; He et al. 2020, 2021). Recent research has demonstrated that fractured bedrock aquifers give rise to elevated concentrations of arsenic and other trace elements in groundwater, and which has been observed in many regions of the world including North America, Africa, and certain regions of Asia (Ayotte et al. 2003; Smedley et al. 2007; Drummer et al. 2015; Ryan et al. 2011 and 2015; Andy et al. 2017). While there are a few case studies in Europe reporting arsenic in fractured bedrock aquifers (Reyes et al. 2015; Morrison et al. 2016), knowledge regarding specific mobilisation processes remains limited.

In Ireland, these hard rock aquifers, or poorly productive aquifers (PPA) underlie 60% of the island and provide an important water source for domestic, commercial and industrial settings (Robins and Misstear 2000). Although not being considered as a source for large public water supplies, they are important for small public group supply schemes and domestic sources, and thus are important in terms of delivering water (and any associated pollutants) via shallow groundwater pathways. Recently, the presence of elevated arsenic (≥ 10 µg L^−1^) has been observed in clusters around Ireland (McGrory et al. 2017).

Total arsenic concentrations fail to provide detailed information regarding the metabolism, toxicity, ecotoxicity and potential mobility in the environment (Michalke 2003). To overcome this, speciation analysis can be undertaken using a hyphenated system (of high-performance liquid chromatography—inductively coupled plasma—mass spectrometry (HPLC-ICP-MS)). However, species redistribution can occur during storage and transport of the samples, influenced by storage time, redox-sensitive parameters, iron concentration, bottle adsorption effects and microbial activity which can have a negative impact on laboratory-based speciation analysis (Leybourne et al. 2014; Ullrich et al. 2016; Kumar and Riyazuddin 2010). An alternative using on-site field speciation of arsenic based on a solid-phase extraction (SPE) cartridge offers advantages over traditional preservation methods with species alternation processes being minimised (Christodoulidou et al. 2012; Ullrich et al. 2016; Bondu et al. 2017).

The aims of this study were to (i) understand the regional geochemistry of arsenic in groundwater of a fractured bedrock aquifer, (ii) understand the mobility of arsenic through speciation studies and (iii) elucidate the geochemical triggers which are responsible for the mobilisation of arsenic within a study site located in the Republic of Ireland as a case study. By understanding arsenic in groundwater at local and regional scales continued effect can be made to assist in reaching the United Nations (UN) Sustainable Development Goals (SDGs), specifically SDG 6: clean water and sanitation.

## Study Site

**Fig. 1:**
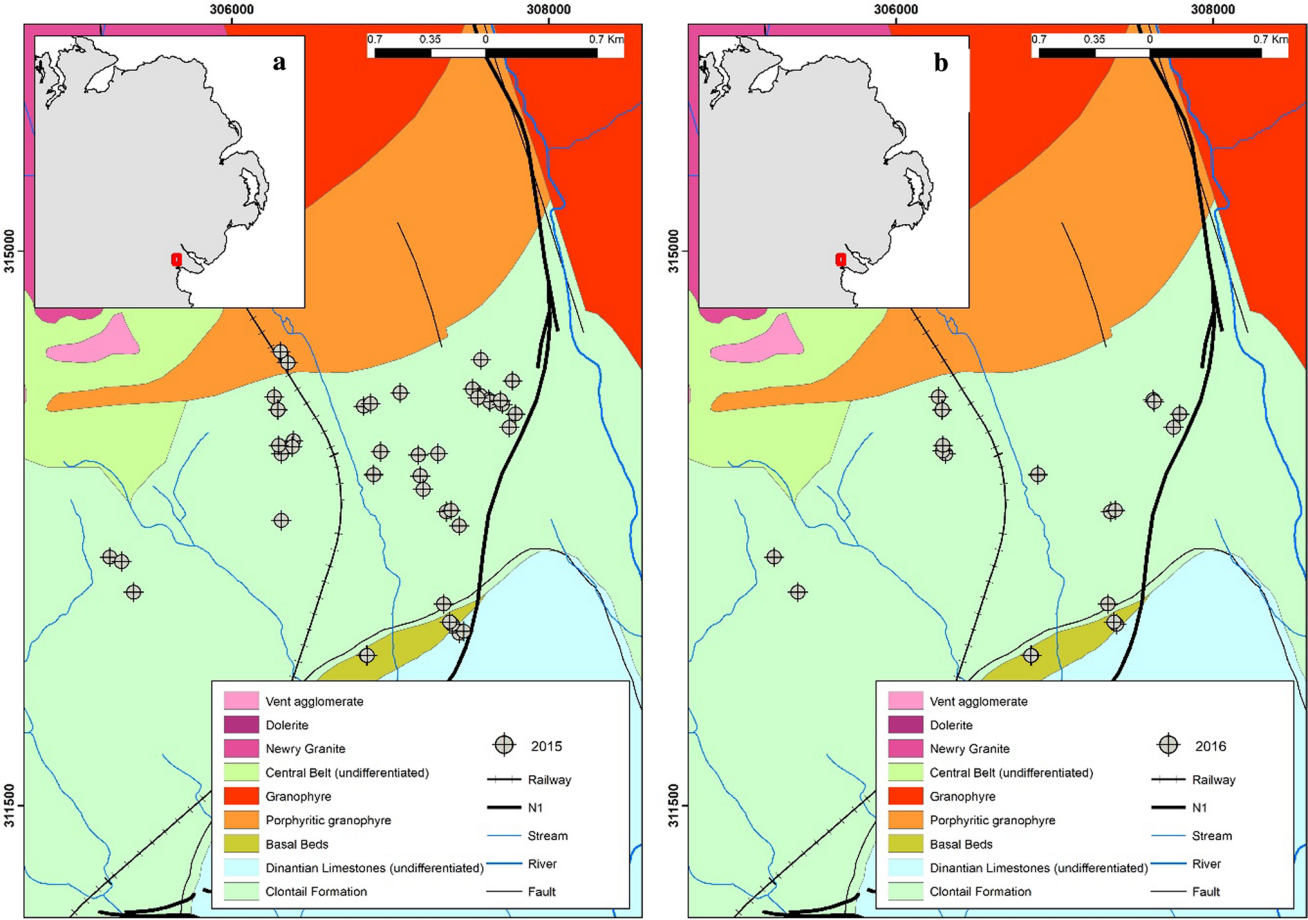
Spatial distribution of groundwater sampling points collected during **a** 2015 (*n* = 43) and **b** 2016 (*n* = 20) overlaid on bedrock geology (1:100 K)

The study site is located north of Dundalk town along the border of the Republic of Ireland and Northern Ireland adjacent to the A1/N1 dual carriageway (Fig. 1a). Most residential homes in the area consist of one-off dwellings with a private well as their primary source of drinking water in addition to an on-site domestic wastewater treatment system (DWWTS). The surrounding area is primarily agricultural (pastures) with forestry to the north (McGrory et al. 2020). The climate of the Ireland is generally mild and humid with changeable weather (temperate oceanic climate). While Ireland has abundant rainfall, generally the east of the country receives less rainfall (750–1000 mm of rainfall a year).

The study area is predominately made up of rocks of the Palaeozoic Southern Uplands-Down-Longford terrane (SUDLT), the Paleogene Slieve Gullion complex with younger intrusives and volcanic rocks to the north, west and east of the study area and Carboniferous sediments found to the south. The SUDLT, which extends across Scotland and Northern Ireland is dominated by Lower Palaeozoic marine sedimentary rocks (lithic arsenites and sandstones) which have undergone low-grade metamorphism (Steed and Morris 1986; Anderson 2004; Lusty et al. 2012; McKinley et al. 2017). The SUDLT bedrock is dominated by well-bedded Ordovician and Silurian turbidite sequences consisting of greywacke sandstone, siltstone and mudstone (Anderson 2004; Lusty et al. 2012). This terrane has been sub-divided into three strike-parallel belts: Northern, Central and Southern Belts (Lusty et al. 2012). Only the Northern and Central Belts of the SULDT occur in the study area (Anderson 2004). Several major strike-parallel faults spaced at 1 and 5 km distance apart, dissect the terrane into a series of fault-bounded tracts (Morris et al. 1986; Anderson 2004; Lusty et al. 2012). The SUDLT contains gold mineralisation including an area in the north-east of Ireland (Clontibret, Monaghan) with Sb-As-Au vein-hosted deposits within Ordovician felsic greywackes (Morris et al. 1986; Steed & Morris 1986; Geraghty 1997; Lusty et al. 2012). Previous research identified a borehole drilled in the greywackes containing elevated levels of both antimony and arsenic in groundwater (As, 9.7–17.76 µg L^−1^; Sb, < 1–132.65 µg L^−1^) (McGrory et al. 2017). The Late Caledonian Newry Igneous Complex intruded into this terrane after closure of the Iapetus Ocean (Cooper and Johnston 2004). During construction of the A1/N1 motorway, which opened in 2007, a 300 m section was excavated showing metasediments of the SULDT in contact with the Newry Igneous Complex porphyritic microgranite along a steep, and locally intensely crushed, contact zone that dips away from the ring-complex (Troll et al. 2008). The ring-complex was formed of silica-rich rocks of fine-grained porphyritic rhyolite and medium-grained porphyritic microgranite (Meade et al. 2010). Surrounding the study area there are also Lower Carboniferous limestones, which include a variety of limestones, shales and dolomites (Geraghty 1997).

Groundwater sampling boreholes and wells penetrate the following geological formations: Porphyritic granophyre (Pg), Dinantian Limestones (undifferentiated) (DIN), Basal Beds (BAS) and Clontail Formation (CL). Most sampling sites are contained within the Clontail Formation which is a calcareous red-mica greywacke, present in Tract 7 of the Central Belt and is made up of grey-green, medium to thick-bedded, coarse and very fine-grained greywackes, with dark grey, thin-bedded, poorly graded, quartzose fine sandstone to siltstone units (Vaughan 1991; McConnell et al. 2001). The CL contains distinctive brown–red coloured biotite indicating a correlation with the Hawick Group on the Southern Uplands in Scotland (Rust 1965; Kemp 1987) and the Inniskeen Formation to the north-west of the study area (McConnell et al. 2001). Based on correlation to the Hawick Group, the formation is thought to be Llandovery to Wenlock in age (McConnell et al. 2001). This formation is bounded to the south by the Salterstown Formation which consists of calcareous white-mica bearing greywacke (Vaughan and Johnston 1992).

There are several Quaternary deposits within the study area with most of the monitoring sites located in areas where the bedrock is overlain by till derived from granites (TGr). Other smaller Quaternary deposits present in this study site include bedrock outcrop or subcrop (Rck), cut-over raised peat (Cut) and alluvium (A) which are present in the north, east and centre of study area. To the west of the monitoring location the bedrock is overlain by till derived from Lower Palaeozoic sandstones and shales (TLPSsS). To the north (in Northern Ireland) quaternary sediments are described as till (diamicton).

The term aquifer is often used for convenience in the Republic of Ireland, even though unaltered bedrock usually does not either store or transmit groundwater. Bedrock below the Republic of Ireland is older than 300 million years. The bedrock is essentially impermeable. However, the Republic of Ireland has considerable groundwater resources. Groundwater is contained in, and moves through, fractures, joints, faults and conduits formed by solution weathering. In other words, water moves underground through the breaks in the rock rather than through the rock matrix. The flow through the network of interconnected narrow and wide breaks in the bedrock could be best described as a groundwater flow system (Institute of Geologists Ireland 2007; Environmental Protection Agency 2013). Irish water supply boreholes are predominantly shallow; few wells are deeper than 120 m. The water table in the bedrock and the overburden is usually close to ground surface, due to elevated rainfall (Collins et al. 2001). The Lower Palaeozoic ‘aquifers’, although fractured, generally do not contain numerous or wide breaks in the rock and are classified as poor aquifers (Geraghty 1997; McConnell et al. 2001). Water can also be obtained from a weathered zone in the shallow bedrock. Sometimes, the top of the weathered bedrock and the bottom of the overburden are indistinguishable, and this zone is commonly termed the transition zone. Generally, the groundwater flow system in the bedrock and transition zone will yield sufficient water to supply a house or a small farm (0.2–0.5 L s^−1^). The yield occasionally is higher in major fracture zones (Geraghty 1997). The yield of shallow wells and boreholes sometimes will depend almost entirely on the groundwater resources in the transition zone, and as a result borehole yields may decease significantly in dry spells as the water table falls. Supplies of water from wells and boreholes are dependent on the resource in the transition zone may be unreliable (Geraghty 1997; McConnell et al. 2001). Much of the Lower Carboniferous clastic rocks (sandstones, siltstones and mudstones) are fractured enough to have a better groundwater flow system, but the yields are still insufficient for the rock types to be regarded as regionally important aquifers (yields of 0.5–3 L s^−1^) (McConnell et al. 2001).

The heterogenous, anisotropic nature of the groundwater flow systems in the bedrock under the Republic of Ireland also means that there can be different flow systems or preferential flow paths at different depths in the rock. These flow systems may be connected to each other or disconnected. Groundwater chemistry and bacteriology can also change with depth. Modern boreholes in the Republic of Ireland are designed and constructed with this consideration in mind (Institute of Geologists Ireland 2007; Environmental Protection Agency 2013). Older, or simply constructed boreholes will often yield water that is an uncontrolled blend of shallow groundwater from the overburden and the transition zone and deep groundwater from one or more open joints, fractures or faults in the deep bedrock (refer to McGrory et al. 2018 for further information).

## Materials and Methods

### Sampling

An extensive water sampling campaign (locations identified as part of a reconnaissance hydrogeological survey in 2014 which is detailed in note 1 of the supplementary information (SI), Fig. S1 and Tables S1-S3. These data were not included as part of geochemical assessment in the present study as they do not contain dissolved arsenic concentrations but were used for initial informative and planning purposes) was conducted in the summer in June 2015 (*n* = 43) and July 2016 (*n* = 20). In June 2015 43 groundwater locations were sampled (Fig. 1a). Subsequently, a smaller subset of these monitoring locations (*n* = 20) were sampled in July 2016 (Fig. 1b) to understand arsenic speciation in groundwater (using both laboratory and field-based methodologies) of boreholes (BH) and dug wells (DW). Samples were collected using appropriate methods such as “clean hands dirty hands” techniques using depth specific low-flow sampling (i.e. 8–10 mbgl) (Puls et al. 1992; Creasey and Flegal 1999; Fitzgerald 1999; Appelo and Postma 2005). To achieve low-flow conditions, a 42 mm stainless steel bladder pump was used with a PCU ProPlus control unit (100 PSI) (In-Situ, UK) (refer to Fig. S2).

Unstable parameters were monitored using the Sheffield low-flow cell (Waterra In-Situ^®^, Shirley, UK) which allows parameters such as pH and Eh to be measured without atmospheric exposure, which are key parameters for understanding aqueous arsenic speciation (Fig. S2 of SI). Probes included temperature (Orion™ 972005MD), Eh (Orion™ 9678BNWP), dissolved oxygen (d-O_2_, Orion™ 083010MD), pH (Orion™ 9107WMMD) and electrical conductivity (EC) (Orion™ 01310MD) monitored using Orion™ 3 and 5 Star meters (Thermo Scientific). Probes were calibrated at each field station using appropriate standards. Relative values for redox potential measured in mV using ZoBell’s solution were corrected for temperature and adjusted to a potential relative to the standard hydrogen electrode (SHE) (Nordstrom 1977; Weight 2008). Groundwater unstable parameters were recorded every five minutes while drawdown/static water level (SWL) was monitored using a dipmeter (OTT Hydrometry KL010 100 m) with drawdown being minimised.

Triplicate groundwater samples were collected once the unstable parameters stabilised and were filtered using 20 cm^3^ BD Discardit™ II PP/PE syringes (VWR, Dublin, Ireland) and Millex®-LCR 25 mm 0.45 µm hydrophilic polytetrafluoroethylene (PTFE) filter (Merck Millipore Ltd., Cork, Ireland) and acidified to pH < 2 using 16 mol L^−1^ HNO_3_ (either using *Optima* HNO_3_ (Fisher Scientific, Dublin) or Romil UpA HNO_3_ (Lennox, Dublin)) in 60 cm^3^ bottles. Additionally, one 250 cm^3^ bottle was used to collect an anion sample. Samples for hydrogen carbonate (HCO_3_^−^) analysis were collected in three 125 cm^3^ bottles and measured in the field. Alkalinity (HCO_3_^−^) was titrated in the field with aliquots of known volume (either 25 cm^3^ diluted with distilled water or 100 mL depending on expected concentration) using a digital titrator (Hach, Model 16,900) using either 0.16 N or 1.6 N H_2_SO_4_ cartridge using bromocresol green–methyl red indicator on the day of collection (Hach-Lange, Dublin, Ireland). All other parameters were measured in the laboratory. Anion samples were not filtered as it was previously demonstrated that there are no significant differences between filtered and unfiltered samples (Daughney et al., 2007). Bottles used for trace metals were washed as per note 2 (SI).

Total dissolved salts (TDS) were estimated from the direct EC measurement of groundwater. This conversion formula is provided in Eq. 1 (Hubert & Wolkersdofer 2015).

1 $${\text{TDS}} = {\text{EC}} \cdot f$$

where f is the conversion factor with 0.69 used here (based on median conductivity of 300–400 µS cm^−1^) (McNeil and Cox 2000; Hem 1985).

### Speciation of Arsenic

Using the technique of field speciation, samples are separated in the field using a solid-phase extraction (SPE) methodology with subsequent separated species determined as ‘total’ concentrations via instrumental analysis in the laboratory for the 2016 monitoring season only (*n* = 20) (O’Reilly et al. 2010); Watts et al. 2010).

For this method, the Varian 500 mg Junior Bond Elut® strong anion exchange (SAX) and 500 mg Junior Bond Elut® strong cation exchange (SCX) cartridges were used (Apex Scientific, Maynooth, Ireland). After the cartridges were conditioned, both were connected in series with a 0.45 µm filter. The sample (25 cm^3^) was passed through the assembly with an Agilent 20 cm^3^ disposable syringe (Apex Scientific, Maynooth, Ireland). The effluent was retained (i.e. arsenite) and both cartridges were separated and 1 M HNO_3_ (Romil, SpA) was passed through the SCX cartridge to collect DMA^V^. For the SAX cartridge, 5 cm^3^ of HOAc (Fluka Analytical, Trace*SELECT*^®^, Sigma-Aldrich, Ireland) was used to collect MA^V^ while iAs^V^ was collected using 1 M HNO_3_.

To perform confirmatory analysis by HPLC-ICP-MS to validate the SPE field speciation procedure (Reilly et al. 2010) a 10 mL sample was collected following filtration (0.45 µm) with the addition of 500 µL of 0.25 M ethylenediaminetetraacetic acid to preserve methylarsenicals (EDTA dipotassium dihydrate, Fluka Analytical, Sigma-Aldrich, Ireland) and stored in the dark at 4 °C (McCleskey et al. 2004; Ujević et al. 2010; Stetson et al. 2021).

### Analytical Measurement

Trace element determination was performed with an ICP-MS (Elan DRCe, Perkin Elmer, Waltham, USA) (Table S4). Due to the presence of chloride in groundwaters, arsenic (^75^As) was measured in dynamic reaction cell (DRC) mode as AsO (m/z 91) with oxygen as the reaction gas in order to correct for the interference at m/z 75 from ^40^Ar^35^Cl^+^ (May and Wiedmeyer 1998). Additionally, due to polyatomic interferences, ^52^Cr, ^56^Fe, ^66^Zn and ^80^Se were analysed in DRC mode using methane as the reaction gas (May and Wiedmeyer 1998). The remaining trace elements (^9^Be, ^11^B, ^27^Al, ^47^Ti, ^51^ V, ^55^Mn, ^59^Co, ^60^Ni, ^63^Cu, ^98^Mo, ^109^Ag, ^111^Cd, ^121^Sb, ^138^Ba, ^184^ W, ^209^Pb and ^238^U) and cations (^24^ Mg, ^23^Na, ^28^Si, ^39^ K, ^43^Ca, and ^88^Sr) were determined in standard mode.

In each analytical batch samples were analysed with certified reference materials (CRMs), blanks (both field and lab) in addition to calibration checks (every 10–14 samples). Full information on linear-working range and LODs of each analyte are documented in Table S5. Triplicate samples taken at each monitoring location were averaged.

A Perkin Elmer Series 200 HPLC system (Perkin Elmer, Waltham, USA) was hyphenated to an ICP-MS and used for confirmatory arsenic speciation analysis. Separation was achieved using the Hamilton^®^ PRP-X100 (4.1 × 250 mm, 5 µm) with NH_4_NO_3_ as the eluting phase (Ammann 2011).

Separation was achieved using a gradient elution method (Table S6) which composed of solvent A (4 mM NH_4_NO_3_) and solvent B (60 mM NH_4_NO_3_) (99.999% Trace Metal Basis, Sigma-Aldrich, Dublin) both adjusted to pH 8.7 (InoLab pH7110) with NH_4_ (Ammonia Solution SpA, Romil, Ireland) (Martínez-Bravo et al. 2001; Watts et al. 2007, 2008). Arsenate (As^V^) and arsenite (As^III^) calibration standards were prepared from 1000 mg L^−1^ standards (Apex Scientific, Maynooth, Ireland), while organicarsenicals standards were prepared from powders of dimethylarsinic acid ((CH_3_)_2_As(O)OH) and disodium methyl arsenate hexahydrate (CH_3_AsNa_2_O_3_•6H_2_O) (Supelco, Sigma-Aldrich, Ireland) with a calibration range of 12.5–100 µg L^−1^.

Analysis of anions was accomplished using a Hach DR 3900™ spectrophotometer (Colarado, USA) and included sulphate (SulfaVer4, 2–70 mg L^−1^ SO_4_^2−^), fluoride (ACCUVAC, 0.02–2.00 mg L^−1^ F^−^) and chloride (LCK311 1–70 mg L^−1^ and 70–1000 mg L^−1^ Cl^−^).

### Quality Control

Overall, CRM (1643e, 1643f, BCR-609 (low-level), BCR-610 (high-level), *Enviro*MAT Ground Water-Low (ES-L-2) and *Enviro*MAT Ground Water-High (ES-H-2)) values fell into the acceptable recovery range of 70–125% for ppb data (Association of Official Analytical Chemists 2002) and an example of CRM recovery for 1643f can be seen in Table S7. The result of this CRM is comparable with published data (Dial et al. 2015; Andy et al. 2017).

Field duplicate samples were generally within ± 10% relative standard deviation (RSD) for all samples. Both field and laboratory blanks showed that metal concentrations were < LOD.

Charge balances were ≤ 10.0%; however, BH-27 (15.3%), DW-7 (15.5%) and BH-46 (12.1%) were higher with the absence of NO_3_ not being measured accounting for this discrepancy based on previous work (McGrory et al. 2020).

### Data and Spatial Analysis

Due to the presence of data below the limit of quantification (LOQ) (i.e. censored data) nonparametric survival analysis procedures were employed to analyse the data (Helsel 2012). This was achieved using the non-detects and data analysis (NADA) macros (version 4.4) with the statistical analysis software Minitab^®^ 17 (available from www.practicalstats.com). Several groups of statistical tests were performed as outlined briefly below:

- Summary statistics—These were computed for different groundwater types (i.e. DW and BH). Due to the presence of censored data (i.e. data reported as < LOQ), these summary statistics were calculated using “robust” regression on order statistics (ROS) using %*Cros* macro (Helsel 2012). Where censoring was above 80%, then the maximum value and censoring rate were presented.
- Correlation and regression—The nonparametric correlation coefficient Kendall’s tau (τ) and test of significance was used to determine the strength of the monotonic relationship between two variables, x and y using the %*Ckend* macro (Helsel and Hirsch 2002; Helsel 2012). Where censoring was ≥ 80% for a variable then it was removed from the analysis. The degrees of relationship (either positive or negative) are denoted as | τ |= 0 (no relationship), | τ |< 0.3 (weak relationship), 0.3 ≤| τ |≤ 0.5 (moderate relationship) and | τ |≥ 0.5 (strong relationship) (Khamis 2008). To access the difference between filtered and unfiltered concentrations of trace elements Kendall’s tau was computed with the non-parametric regression line associated with Kendall’s tau, the Akrita-Theil-Sen (ATS) line using the %*ATS* macro (Helsel 2012). All tests were computed at the 0.05 significance level.
- Multivariate statistical analysis (MSA)—To account for censored data, censored multivariate techniques were employed based on ordinal methods using the %*ordranks* macro (Helsel 2012). Subsequent ranks were used as input for principal component analysis (PCA) and the extraction methods of Varimax rotation and Kaiser normalisation were applied to interpret geochemical data using IBM® SPSS® StatisticsV25 (Wu et al. 2020; Li et al. 2019). Both Kaiser’s measure of sampling adequacy (KMO) and Bartlett’s test of sphericity were performed to assess the sampling adequacy for their suitability for PCA which showed data were suitable for PCA (Bartlett 1950; Kaiser and Rice 1974). Principal components (PCs) with an eigenvalue greater than one were retained (Kaiser 1960). Hierarchical cluster analyses (HCA) were performed on ranked data in Q mode (variables) using Euclidean distance measures with Ward’s methods (Nnane 2011; Wangkahad et al. 2017). HCA was also performed on ranked data in R mode (sampling sites) to access spatial clustering of parameters using ArcGIS 10.6.1 (projection, TM-65; datum, D-TM65).

### Aqueous Geochemical Modelling

Eh–pH diagrams were constructed for the system As–O–H using the ‘Act2’ program with the Lawrence Livermore National Laboratories (LLNL) thermodynamics database ‘thermo.tdat’ in Geochemist’s Workbench® (Release12.0, Student Edition) (Bethke and Yeakel 2018). Temperature was set at 25 °C, pressure at 1 bar and arsenic activity set at 10^–6^ M (Lu and Zhu 2011).

Saturation indexes (SI) for minerals were calculated using PHREEQCI V3.4 (Appelo and Postma 2005) using the WATEQ4F database (Ball and Nordstrom 1991).

## Results and Discussion

### Physicochemical Parameters

**Fig. 2:**
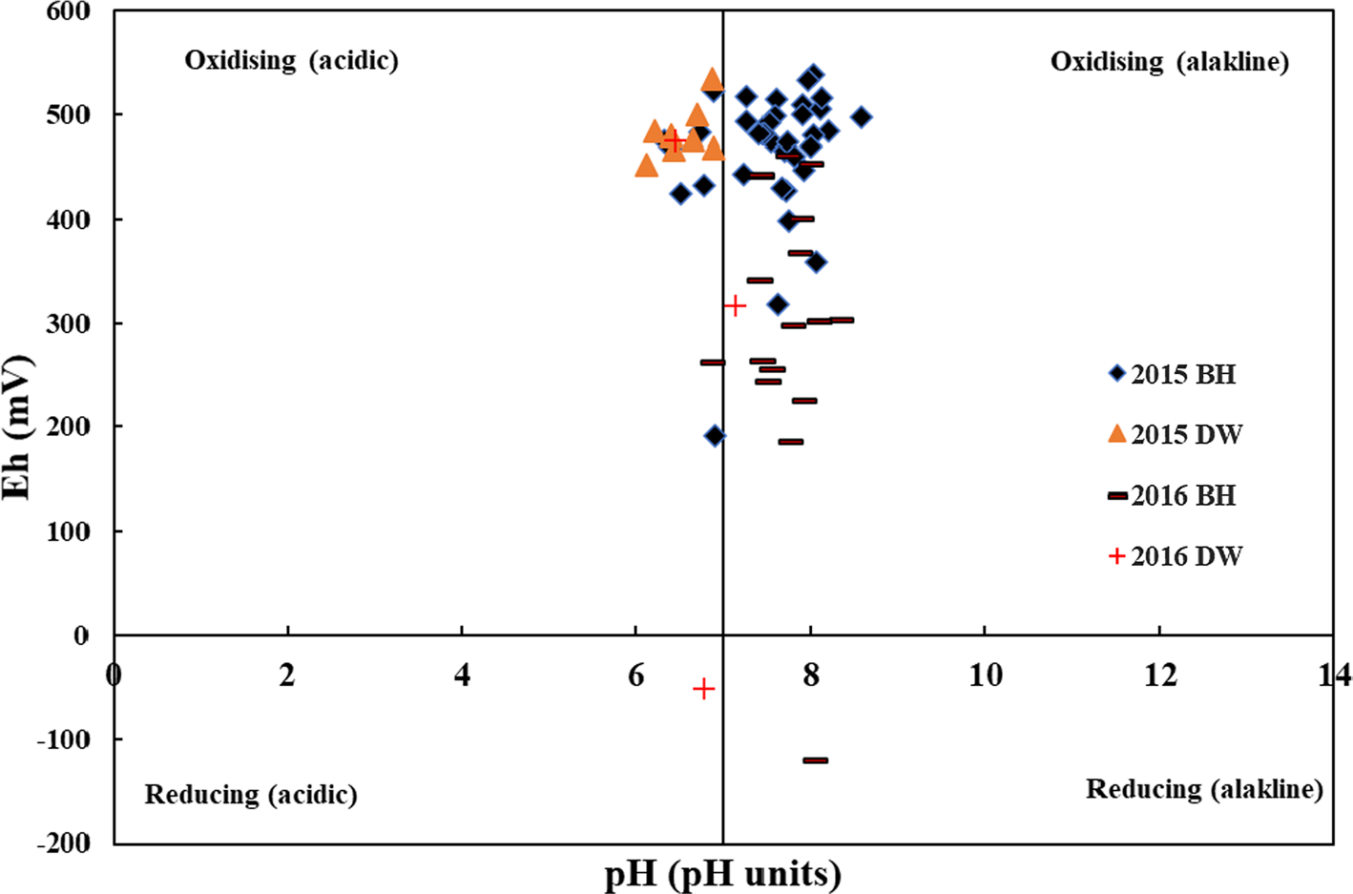
Graphical representation of oxidising to reducing and acidic to alkaline environments for groundwater data in 2015 and 2016. For reference, both DW-17 and BH-40 are at the bottom of the figure

Statistical summaries of data measured in 2015 are shown in Table 1 (2016 data presented in Tables S8-S9). Eh values are indicative of more oxidising conditions in shallow dug wells (range—2015, 452.85–534.8 mV; 2016,  52–476 mV) when compared to boreholes (range—2015, 191.6–538.3 mV; 2016, -121.5–460.3 mV). However, some mildly reducing conditions were observed in 2016 in both the wells, DW-17 (Eh-52.1 mV, pH 6.7) and BH-40 (Eh -121.5 mV, pH 8.0). Groundwaters from boreholes were acidic to alkaline with pH ranging from 6.3 to 8.6 with dug wells being acidic ranging from 6.1 to 6.9. In comparison, pH values in 2016 increased where groundwater in boreholes was slightly acidic to alkaline with pH ranging from 6.9 to 8.3 with dug wells being acidic to near-neutral ranging from 6.5 to 7.1. The more acidic nature of surface wells may reflect more surface weathering of the acidic soil. Previous work in this area has also identified that surface wells were contaminated with elevated nitrate arising from inorganic fertilisers (McGrory et al. 2020). Generally, groundwaters were described as alkaline oxidising (or oxic-alkali) for boreholes and acidic oxidising (or oxic-acidic) groundwaters for the shallower dug wells (Fig. 2). While some boreholes were described as acidic oxidising, all of these shallower dug wells are categorised as low-arsenic wells. Previous studies in Quebec have also demonstrated shallow wells displaying more oxidising conditions when compared to bedrock wells (Bondu et al. 2017). The Eh–pH conditions overlap with results obtained for both shallow and deep groundwaters in western Ireland (Gilligan et al. 2016). These oxidising conditions are consistent with the concentrations of d-O_2_ measured in 2016 (4.17 ± 6.88 mg L^−1^ for BHs and 5.23 ± 3.06 mg L^−1^ for DWs). Only three sites have low d-O_2_ measurements (BH-20, 0.22 mg L^−1^; BH-47 0.12 mg L^−1^; and BH-25, 0.485 mg L^−1^), representative of a suboxic redox state (McMahon and Chapelle 2008). As other wells had d-O_2_ ≥ 0.5 mg L^−1^, Mn ≤ 50 µg L^−1^, and Fe ≤ 100 µg L^−1^_,_ the redox couple present in these groundwaters is likely O_2_ reduction (Thomas 2007; McMahon and Chapelle 2008). However, for other wells where Mn ≥ 50 µg L^−1^ and/or Fe ≥ 100 µg L^−1^ with d-O_2_ ≥ 0.5 mg L^−1^ this in characteristic for a mixed redox state (Thomas 2007; McMahon and Chapelle 2008). This large variation of d-O_2_ for BHs may reflect a combination of different redox states possibly though blending of groundwater (refer to Study Site section).

**Table 1:** Statistical summary of hydrochemistry data in bedrock boreholes (BH, *n* = 35) and dug wells (DW, *n* = 8) sampled in 2015

Variable	Groundwater Type	Mean	SD	Min	Q1	Median	Q3	Max	Cen (%)	Limit	% > Limit
Depth (m)	BH	76.6	35.8	9.10	49.3	79.4	104.0	148.8	0.0	–	NA
	DW	4.3	1.8	2.0	2.8	3.8	6.0	7.0	0.0		NA
SWL (m)	BH	6.4	5.8	0.1	2.7	5.2	8.1	32.7	0.0	–	NA
	DW	2.5	1.5	0.5	1.1	2.6	3.2	5.2	0.0		NA
Temp (°C)	BH	11.3	1.2	9.8	10.3	11.4	11.7	16.3	0.0	–	NA
	DW	10.4	0.9	9.7	9.8	10.0	10.8	12.5	0.0		NA
Cond (µS cm^−1^)	BH	388.3	220.4	87.7	239.5	374.0	477.7	1405.0	0.0	2500	0.0
	DW	440	320	134	169	399	619	1044	0.0		0.0
TDS (mg L^−1^)	BH	266.8	152.8	60.5	159.9	258.1	329.6	969.4	0.0	-	NA
	DW	303.6	221.1	92.2	116.5	275.4	427.1	720.0	0.0		0.0
pH	BH	7.5	0.5	6.3	7.3	7.65	7.96	8.6	0.0	≥ 6.5 & ≤ 9.5	5.7
	DW	6.5	0.3	6.1	6.3	6.55	6.83	6.9	0.0		50.0
Eh (mV)	BH	463.1	66.3	191.6	442.7	480.8	499.9	538.3	0.0	–	NA
	DW	483.2	25.0	452.9	468.0	478.5	496.1	534.8	0.0		NA
Cl^−^ (mg L^−1^)	BH	19.7	8.5	1.4	13.8	18.4	24.7	37.9	0.0	250	0.0
	DW	52.5	27.0	76.5	12.2	15.3	17.1	59.9	0.0		0.0
SO_4_^2−^ (mg L^−1^)	BH	15.7	12.2	8.0	10.0	13.0	17.0	81.0	0.0	250	0.0
	DW	12.2	3.4	9.0	10.0	11.0	15.0	19.0	0.0		0.0
F^−^ (mg L^−1^)	BH	0.2	0.4	0.1	0.1	0.1	0.2	2.5	0.0	1.5	2.8
	DW	0.1	0.0	0.1	0.1	0.1	0.2	0.2	0.0		0.0
Alk (mg L^−1^)	BH	119.9	45.8	33.4	76.6	120.1	159.6	203.3	0.0	-	NA
	DW	101.9	70.5	33.7	38.6	85.6	173.5	216.9	0.0		NA
Be (µg L^−1^)	BH	–	–	–	–	–	–	0.2	97.2	-	NA
	DW	–	–	–	–	–	–	–	100		NA
B (µg L^−1^)	BH	10.3	4.3	6.3	7.3	9.0	11.8	23.5	0.0	1000	0.0
	DW	14.0	7.9	6.5	7.2	12.7	17.0	30.7	0.0		0.0
Al (µg L^−1^)	BH	3.5	8.6	0.3	0.7	1.4	2.9	51.3	0.0	200	0.0
	DW	6.0	6.9	0.7	1.3	2.4	11.9	19.4	0.0		0.0
Ti (µg L^−1^)	BH	0.8	0.9	0.2	0.5	0.8	1.0	5.9	2.8	–	NA
	DW	0.7	0.3	0.4	0.5	0.6	1.0	1.2	0.0		NA
V (µg L^−1^)	BH	1.7	3.4	0.1	0.2	0.5	1.3	15.8	5.7	–	NA
	DW	0.7	0.4	0.4	0.5	0.6	0.9	1.4	0.0		NA
Cr (µg L^−1^)	BH	0.2	0.6	0.0	0.0	0.0	0.1	3.3	65.7	50	0.0
	DW	0.1	0.1	0.0	0.0	0.1	0.2	0.2	37.5		0.0
Mn (µg L^−1^)	BH	20.6	103.2	0.0	0.2	0.3	1.5	611.0	8.6	50	2.8
	DW	26.4	36.2	0.0	0.4	2.5	60.5	89.5	12.5		37.5
Fe (µg L^−1^)	BH	1132	6634	0	0	1	5	39,257	45.7	200	5.7
	DW	53.1	112.5	0.1	0.4	3.5	66.8	321.9	12.5		12.5
Co (µg L^−1^)	BH	0.04	0.1	0.0	0.0	0.0	0.0	0.7	71.4	-	NA
	DW	0.2	0.1	0.0	0.1	0.1	0.3	0.3	37.5		NA
Ni (µg L^−1^)	BH	1.2	0.5	0.4	0.8	1.1	1.6	2.6	2.8	20	0.0
	DW	1.2	0.9	0.5	0.6	1.7	2.2	2.6	0.0		0.0
Cu (µg L^−1^)	BH	0.9	0.8	0.1	0.4	0.5	1.0	3.3	5.7	2000	0.0
	DW	7.0	14.3	0.3	0.6	1.7	4.8	42.0	0.0		0.0
Zn (µg L^−1^)	BH	2.9	3.4	0.3	1.0	2.1	2.9	14.1	0	5000	0.0
	DW	20.5	27.5	0.6	0.9	2.6	48.8	67.7	0.0		0.0
**As (µg L**^**−1**^**)**	**BH**	**11.3**	**12.6**	**0.1**	**1.4**	**7.2**	**19.4**	**51.3**	**8.6**	**10**	**45.7**
	**DW**	**0.6**	**1.3**	**0.0**	**0.0**	**0.2**	**0.5**	**3.7**	**25.0**		**0.0**
Se (µg L^−1^)	BH	0.5	0.9	0.00	0.0	0.1	0.5	4.0	37.1	10	0.0
	DW	0.1	0.1	0.0	0.1	0.1	0.2	0.2	37.5		0.0
Mo (µg L^−1^)	BH	1.8	2.0	0.1	0.2	1.5	2.4	8.1	17.1	–	NA
	DW	0.4	0.1	0.3	0.3	0.4	0.5	0.5	62.5		NA
Ag (µg L^−1^)	BH	–	–	–	–	–	–	0.1	97.1	–	NA
	DW	–	–	–	–	–	–	0.2	87.5		NA
Cd (µg L^−1^)	BH	–	–	–	–	–	–	0.0	97.1	5	0.0
	DW	–	–	–	–	–	–	0.0	87.5		0.0
Sb (µg L^−1^)	BH	0.4	0.7	0.0	0.0	0.1	0.3	3.0	40.0	5	0.0
	DW	–	–	–	–	–	–	0.3	75.0		0.0
Ba (µg L^−1^)	BH	43.5	67.7	0.1	0.6	5.2	77.5	285	0.0	500	0.0
	DW	11.2	10.4	3.0	6.4	7.6	12.9	35.8	0.0		0.0
W (µg L^−1^)	BH	0.3	1.4	0.0	0.0	0.00	0.0	8.0	77.1	–	NA
	DW	–	–	–	–	–	–	–	100		NA
Pb (µg L^−1^)	BH	0.06	0.1	0.0	0.0	0.0	0.2	0.5	77.1	10	0.0
	DW	–	–	–	–	–	–	0.3	87.5		0.0
U (µg L^−1^)	BH	1.6	2.4	0.0	0.2	0.6	2.4	9.3	20.0	30	0.0
	DW	0.2	0.2	0.0	0.0	0.1	0.2	0.8	25.0		0.0
Mg (mg L^−1^)	BH	11.8	8.7	0.6	5.1	9.6	17.7	34.4	0.0	50	0.0
	DW	9.4	7.1	2.1	3.4	7.6	16.5	20.6	0.0		0.0
Si (mg L^−1^)	BH	6.1	1.9	1.0	4.8	5.7	6.9	11.3	0.0	–	NA
	DW	5.9	1.7	3.9	4.6	5.7	7.0	9.1	0.0		NA
Ca (mg L^−1^)	BH	41.6	19.1	11.8	26.5	39.8	56.7	73.7	0.0	200	0.0
	DW	52.5	33.9	14.1	18.5	55.5	80.9	95.0	0.0		0.0
Na (mg L^−1^)	BH	13.6	5.7	2.9	9.7	13.1	16.3	35.9	0.0	200	0.0
	DW	24.6	31.3	8.8	9.8	11.4	27.4	100.0	0.0		0.0
Sr (mg L^−1^)	BH	0.3	0.3	0.0	0.1	0.2	0.5	1.1	0.0	–	NA
	DW	0.1	0.0	0.0	0.1	0.1	0.2	0.3	0.0		NA
K (mg L^−1^)	BH	1.9	2.9	0.4	1.1	1.4	1.8	18.1	0.0	5	2.8
	DW	2.4	2.2	0.7	1.1	1.5	3.2	7.5	0.0		12.5

Bold values indicate arsenic data

Conductivity was higher in shallow surface dug wells compared to deeper boreholes with two wells (DW-11 and BH-23) having high conductivities (> 1000 µS cm^−1^) in 2015 and were marginally lower in 2016. The SWLs were < 9 m for boreholes, however some in the 60–120 m depth category were sometimes greater than 10 m. The deepest SWL was recorded in BH-39 in both years (2015, 32.76 m; 2016, 30.15 m). The shallowest BH was BH-18 with a SWL of 0.04 m in 2015. A linear regression showed that GWLs in both years (*n* = 19) are positively correlated (*n* = 19, *τ* = 0.54, *p* = 0.001) with GWLs from 2016 being approximately 2.9% higher when compared to 2015.

### Major-ion Geochemistry and Hydrogeochemical Facies

**Fig. 3:**
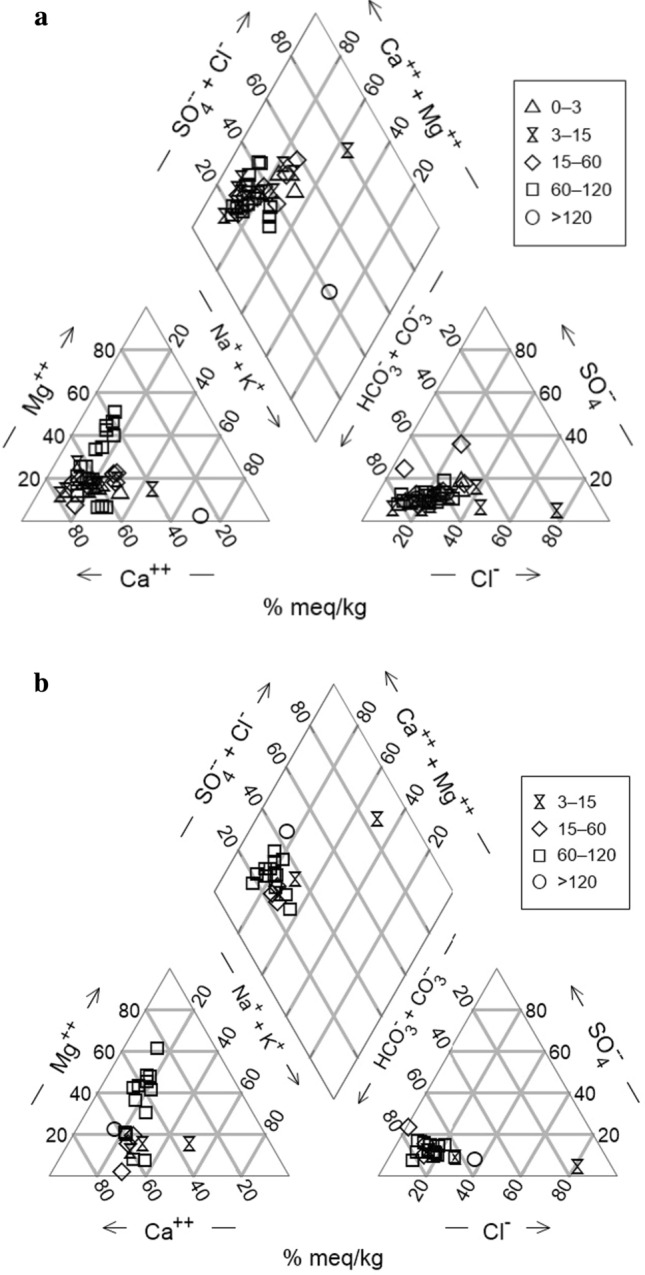
Piper diagram for **a** 2015 and **b** 2016 data. Samples are classified by groundwater well depth category (m)

According to the Piper diagram (He and Li 2020; Fig. 3a–b), hydrochemical facies of groundwater are dominated by Ca-Mg-HCO_3_ and Ca-HCO_3_ indicating mainly recharged groundwater for both shallow and deep groundwater wells. This reflects the calcareous nature of the Clontail Formation and Dinantian Limestones which most boreholes are transected in.

Most sites are classified as recharging waters (Ca-HCO_3_^−^) (Fig. S3a-b) using Chadha’s diagram (Chadha 1999). For 2015 some groundwaters are governed by ion-exchange (Na-HCO_3_^−^) or reverse ion-exchange waters (Ca–Mg–Cl). In 2016 one well (DW-11) falls into the seawater (Na-Cl) category. Inadequate wastewater treatment may be responsible for the elevated concentrations of both Na and Cl in this surface well (DW-11). In 2015 this well had a Cl^−^ concentration of 236 mg L^−1^ (average ± SD in 2015 for DWs was 52 ± 27 mg L^−1^) while Na was 99.8 (average ± SD in 2015 for DWs was 24.6 ± 31.3 mg L^−1^). The Gibbs diagram (Gibbs 1970) showed most samples in this locality lie within rock weathering dominance (Fig. S4a–d). A small number of samples lie in the evaporation dominance category and these wells are associated with high Cl^−^ and Na^−^ concentrations (DW-11) showing localised contamination. Thus, the main processes contributing to groundwater geochemistry in this area is rock weathering, i.e. the interaction of groundwater with aquifer material. Aquifer rock weathering facilitates in the geochemical process that soluble salts and minerals that can become incorporated into groundwater (Talib et al. 2019). In addition, the longer residence times associated with rock–water interactions also aids in this mineral dissolution (Selvakumar et al. 2017; Talib et al. 2019).

All anions and cations were below the regulated concentrations except for F and K where maximum concentration was 2.46 mg L^−1^ (BH-58) and 18.08 mg L^−1^ (BH-06), respectively. Fluoride does not show many correlations with major anions, but a weak negative correlation exists with K (*τ* = -0.24) (unless otherwise stated all correlations coefficients are for 2015 data with those denoted by * are not significant at the 5% significance level). Several ions are correlated with depth including weak positive relationships for SO_4_^2−^ (*τ* = 0.19*), F^−^ (*τ* = 0.25), Mg (*τ* = 0.25), Na (*τ* = 0.23), and Si (*τ* = 0.22). Different levels of strength of the monotonic relationship are seen for several ions: strong positive relationships (SO_4_^2−^ and Sr (*τ* = 0.50), HCO_3_^−^ and Mg (*τ* = 0.54), HCO_3_^−^ and Ca (*τ* = 0.54), HCO_3_^−^ and Sr (*τ* = 0.57), SO_4_^2−^ and Mg (*τ* = 0.56), Si and Na (*τ* = 0.51), and Sr and Mg (*τ* = 0.65)), moderate positive relationships (Cl and Na (*τ* = 0.30), Ca and Cl (*τ* = 0.44), Ca and Na (*τ* = 0.30), Ca and K (*τ* = 0.39), Ca and Si (*τ* = 0.30), Mg and Ca (*τ* = 0.30), and Si and K (*τ* = 0.30)), and weak positive relationships (Cl and Mg (*τ* = 0.25), Cl and Sr (*τ* = 0.24), SO_4_^2−^ and Si (*τ* = 0.23), SO_4_^2−^ and Ca (*τ* = 0.25), SO_4_^2−^ and Na (*τ* = 0.27), SO_4_^2−^ and K (*τ* = 0.28), Ca and Sr (*τ* = 0.27), Mg and Si (*τ* = 0.17*), and HCO_3_^−^ and K (*τ* = 0.21)). Sr shows a weak positive correlation with pH (*τ* = 0.23) while K shows a weak negative correlation with depth (*τ* =  0.14*). Conductivity shows a strong positive correlation with Ca (τ = 0.68), HCO_3_^−^ (τ = 0.63), moderate positive correlation with SO_4_^2−^(τ = 0.45), Cl (τ = 0.48), Mg (τ = 0.46), Na (τ = 0.37), Sr (τ = 0.40) and K (τ = 0.31), and weak positive correlation for Si (τ = 0.26). The strong positive correlation with Ca and HCO_3_^−^ (τ = 0.54) also indicates that these ions are the major ones resulting from rock or soil weathering from their patent aquifer material in this study location. Given the correlation of HCO_3_^−^ with Ca and Mg this is indicative of the dissolution of carbonate minerals, i.e. calcite and dolomite (Talib et al. 2019). The molar ratio for calcite and dolomite dissolution is 1:2 and 1:4, respectively (Appelo and Postma 2005). While the molar ratio deviates from the 1:1 line so dissolution of these minerals does not fully account for these cations in groundwater, however some of the data points have a ratio of 1:2 and 1:4. The molar ratio of Na/Cl of nearly 1:1 for these samples is indicative of halite dissolution (Talib et al. 2019; Li et al. 2016, 2018). However, ion exchange processes may also be occurring (as Na/Cl < 1) while contribution of extra Na may result from silicate weathering given that some samples lie above the equiline of 1:1 (as Na/Cl > 1) (Juen et al. 2015; Ren et al. 2021). Indeed, given the correlation of Si with cations (Na, Ca, Mg, and K) this further illustrates silicate weathering in the groundwater (Montcoudiol et al. 2014; Bondu et al. 2017). This is supported by the fact that groundwaters are undersaturated with respects to silicate minerals such as anorthite, but the extent of this is not fully known given that PHREEQC calculations indicate that calcite is both under and oversaturated in samples (discussed further in Thermodynamic calculations section).

### Trace Elements

Most trace elements show a linear relationship of filtered (dissolved) to unfiltered (total) concentrations. In these groundwaters, indicating that most trace elements exist in dissolved form and are negligible with regards to particulate form (Table S10). However, some occur in greater proportions in particulate form such as for Al, Fe, Mn, Ti and Pb. For the rest of the discussion, unless otherwise stated, interpretations will be made with reference to dissolved aqueous concentrations. In terms of water quality exceedances (besides arsenic) the greatest occurred for Fe, Mn and U. While these exceedances are evident for Fe and Mn in both shallow and deep groundwater, exceedances for U and As only occurred in deeper bedrock boreholes.

Detected trace elements were found at low concentrations in groundwater (with corresponding lower concentrations measured in dug wells) with Al, As and Ba occurring at higher concentrations. As seen elsewhere, the concentrations of Sb, Cu, Mo, Cd, Se and Be rarely exceed regulatory limits (DeSimone et al. 2009). When Fe and Mn are reported as occurring at higher concentrations, this is generally a result of an outlier. For example, one sampling location in 2015 (BH-66) recorded Mn and Fe concentrations at 611 and 39,257 µg L^−1^, respectively. This anomalously high value in comparison with the lower values in the surrounding area arises from the reductive dissolution of Fe and Mn oxyhydroxides (reducing conditions noted while sampling) as the well is not currently in active use. Indeed, concentrations of these trace elements are usually low with higher concentrations found in private wells elsewhere (DeSimone et al. 2009; Homoncik et al. 2010; McGrory et al. 2018).

Both Mn and Fe can be derived from the weathering of multiple common bedrock minerals which includes silicates, oxides, carbonates and sulphides (Bondu et al. 2017). Both Fe and Mn have high particulate concentrations when compared to other trace elements. These particulate forms of Fe and Mn may have sorbed oxyanions and other trace elements with the sample acidification step promoting their dissolution into dissolved Fe and Mn (Plant et al. 2003). Particulate concentrations for most trace elements are negligible and reflects the natural filtration of the groundwater through the aquifer (Plant et al. 2003).

Several parameters show a positively weak to strong correlation with depth data including pH (*τ* = 0.51), HCO_3_^−^ (*τ* = 0.21), temperature (*τ* = 0.34), F (*τ* = 0.25) and several oxyanions (As (*τ* = 0.52), Mo (*τ* = 0.51), Sb (*τ* = 0.31) and U (*τ* = 0.40)). While Fe and Mn show a strong positive correlation (*τ* = 0.63), both show weak-to-moderate negative correlations with Eh, pH and depth. Several oxyanions (Mo, Se, Sb, U, and HCO_3_^−^) also display negative weak-to-moderate correlations with Fe and Mn. Positive correlation of Fe and Mn have been observed for metasedimentary geologic units (Ayotte et al. 1999), crystalline bedrock (Johnson et al. 2017) and mixed bedrock (Homoncik et al. 2010). Given the strong positive relationship between Fe and Mn, they are expected to originate from a common source. Similar trends with Eh, pH and depth are observed with B, Cu, Co and to a lesser extent with Al, V, Cr and Ni. Different levels of strength of the monotonic relationship are evident for several transition series trace elements: moderate positive relationships (Cr and V (*τ* = 0.37), Cu and Zn (*τ* = 0.31), Mn and V (*τ* = 0.40*), and weak positive relationships (Cr and Ti (*τ* = 0.22), Co and Mn (*τ* = 0.18), V and Ti (*τ* = 0.24), Ti and Co (*τ* = 0.19), V and Co (*τ* = 0.14), Co and Ni (*τ* = 0.29), Co and Cu (*τ* = 0.16), Ni and Cu (*τ* = 0.21) and Pb and Zn (*τ* = 0.19)). While not a transition series trace element, B shows a strong positive correlation with Ni (*τ* = 0.50), Ca (*τ* = 0.56), moderate positive correlation with Na (*τ* = 0.31) and K (*τ* = 0.39) and weak positive correlation with V (*τ* = 0.28), Co (*τ* = 0.24), Cu (*τ* = 0.24) and Si (*τ* = 0.26).

### Arsenic Geochemistry and Speciation

Arsenic was the most frequently detected parameter above the regulatory limit (10 µg L^−1^) with 45.7% and 70.5% of samples having concentrations above the limit in 2015 and 2016 for boreholes with a maximum value recorded at 73.95 µg L^−1^ in 2016. Previous work has identified that arsenic concentrations in this area to the north-west of the study site approach 139 µg L^−1^ (McGrory et al. 2017). Arsenic was detected at lower concentrations in dug wells with the maximum concentration reported at 3.7 µg L^−1^ in 2015, comparable to observations noted in a bedrock aquifer in Quebec, Canada where arsenic concentrations in shallow wells did not exceed 4.1 µg L^−1^ (Bondu et al. 2017). Negligible arsenic concentrations were reported in dug wells as no anthropogenic contamination was noted in this area (McGrory et al. 2020). Anthropogenic arsenic contamination (or surface contamination) would show higher arsenic concentrations in dug wells and would mask the positive correlation of arsenic and depth present in this study.

Groundwaters with elevated arsenic (≥ 10 µg L^−1^) were usually associated with an Eh of > 390 mV (lower Eh in 2016 ~  > 120 mV) and near-neutral to alkaline pH, depth > 70 m and low concentrations of dissolved Fe (< 2 µg L^−1^) and dissolved Mn (< 1.5 µg L^−1^). One borehole (BH-61) had a low arsenic concentration in 2015 (8.1 µg L^−1^), but a moderate one in 2016 (15.4 µg L^−1^) with Fe and Mn measured at 30.5 µg L^−1^ and 8.5 µg L^−1^ in 2016. However, total concentrations of Fe measured markedly higher at 1588.6 µg L^−1^ with Mn at 28.1 µg L^−1^ showing that low-arsenic concentrations can occur with elevated Fe and/or Mn concentrations. Low-arsenic wells usually had a lower pH of 6.4–7.4 with a variable Eh. While considered low-arsenic wells, both BH-46 and BH-70 had a pH of 7.9 and 8.3 with arsenic concentrations of 6.04 µg L^−1^ and 8.12 µg L^−1^, respectively (2016). This illustrates that even at low concentrations arsenic may still be mobilised through desorption processes. It has been demonstrated that wells with high arsenic concentrations are generally associated with concentrations of iron > 100 ppb and are have reducing conditions (Erickson et al. 2019). Given the low Fe concentrations, this also agrees with the oxic-alkali nature of the groundwater in the present study. Furthermore, the level of arsenic contamination is generally lower in oxic-aquifers compared to anoxic aquifers (Masuda 2018).

**Fig. 4:**
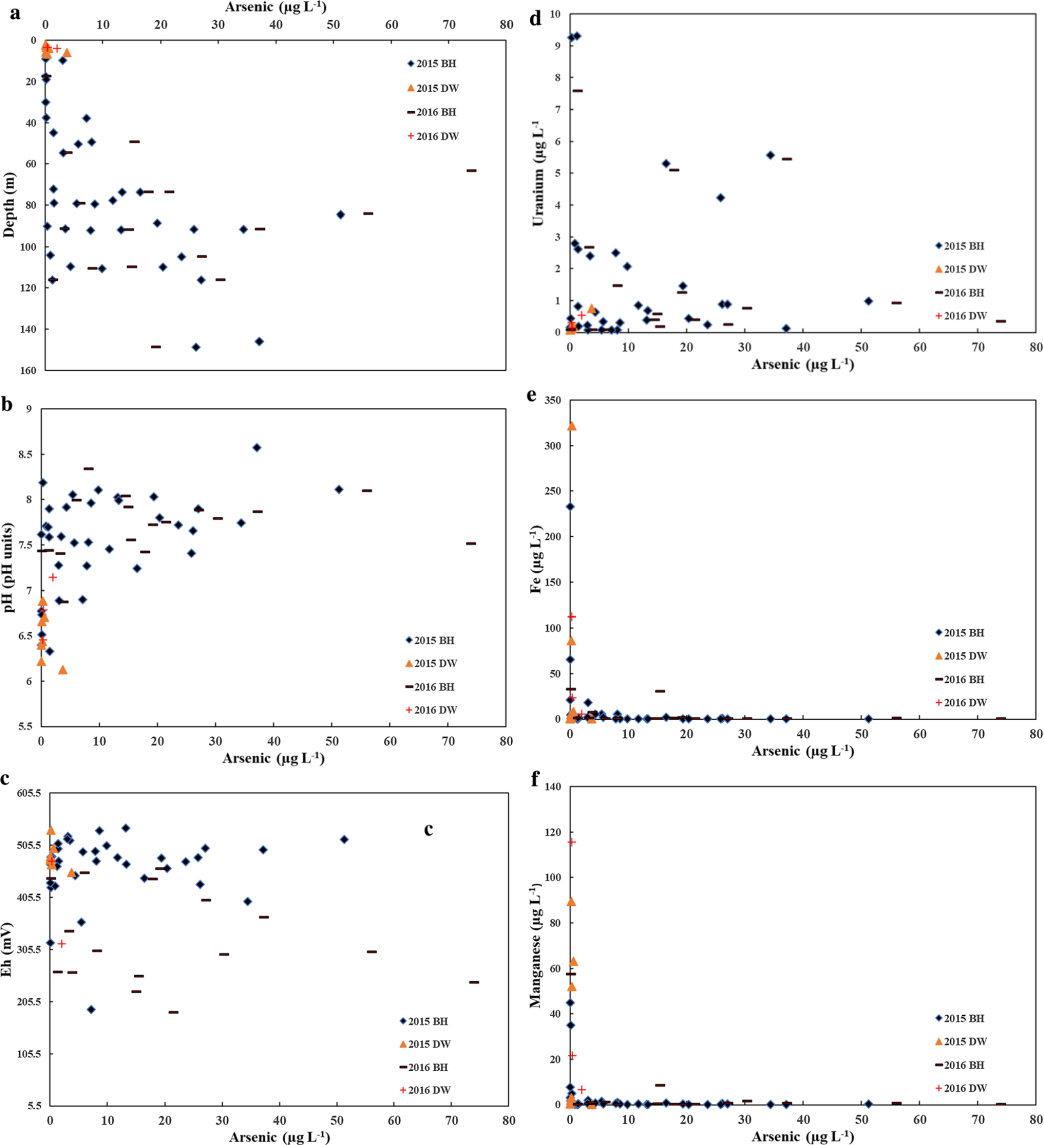
Biplot for arsenic and **a** depth, **b** pH, **c** Eh, **d** uranium, **e** Fe and **f** Mn. Note that BH-66 was removed for both Fe and Mn for 2015 data

In comparison, elevated arsenic concentrations were mainly distributed between 70 and 120 m depth (Fig. 4a). A small proportion of boreholes with elevated arsenic concentrations occur at a depth > 120 m. For low-arsenic concentration boreholes (i.e. ≤ 10 µg L^−1^) these occurred within several depth categories. Elevated arsenic is observed at pH ~  > 7.5 (up to 8.57 (Fig. 4b)) and > 390 mV (Fig. 4c). However, another group of elevated arsenic wells occurs between 200 and 310 mV. This may be indicative of mixed redox state groundwater thereby suggesting a mixing of groundwater. Elevated arsenic at an alkaline pH in addition to lack of relationship with Eh has been shown to occur in oxidising aquifers (Rango et al. 2013). Indeed, alkaline waters favour the release of arsenic (Shaji et al. 2021). Elevated arsenic concentrations show U concentrations below 6 µg L^−1^; however, elevated U can be seen in low-arsenic wells (Fig. 4d). Elevated arsenic concentrations were observed with low Fe and Mn while elevated Fe and Mn were seen with low-arsenic concentrations (Fig. 4e–f). This is seen with a moderate negative correlation of arsenic with Fe and Mn.

Concentrations of total and dissolved arsenic from both study years were highly correlated (2015, *τ* = 0.93, *p* = 0.0; 2016, *τ* = 0.96, *p* = 0.0). A linear regression showed that the unfiltered arsenic concentrations were approximately 12.4% and 2.2% higher than the filtered samples in 2015 and 2016, respectively, indicating that the particulate form of arsenic was negligible in these groundwaters with similar results been observed in private bedrock boreholes elsewhere (Peters et al. 1999; Ayotte et al. 2003; Kim et al. 2003). A linear regression demonstrated that concentrations of arsenic concentrations sampled in both years (*n* = 19) are positively correlated (*n* = 19, *τ* = 0.87, *p* ≈ 0.000) with samples obtained in 2016 being approximately 8.1% higher. Similar results have been observed for arsenic in private bedrock boreholes in New Hampshire (Ayotte et al. 2003). The absolute value of intra-well differences ranged from 0.06–8.1 µg L^−1^, with a median difference of 1.44 µg L^−1^.

Several parameters show an association with arsenic in groundwaters including strong positive correlations (depth (*τ* = 0.52), Mo(*τ* = 0.51)), moderate positive correlations (pH (*τ* = 0.45), temperature (*τ* = 0.37), HCO_3_^−^ (*τ* = 0.37), SO_4_^2−^ (*τ* = 0.31), Sb (*τ* = 0.44), U (*τ* = 0.32), Sr (*τ* = 0.47) and Mg (*τ* = 0.36)), and weak positive correlations (conductivity (*τ* = 0.22), Se (*τ* = 0.20) and Ba (*τ* = 0.23)). The previous correlations with several oxyanions (e.g. Se, Mo and Sb) were reported in previous studies (McGrory et al. 2020) with the degree of the monotonic relationship being stronger in this present study. As previously mentioned, both Fe and Mn show a moderate negative relationship with arsenic (*τ* =  0.40 and *τ* =  0.36, respectively), this is also obvious for Cu which displays a weak negative relationship (τ =  0.27). The strong correlation of arsenic and depth is also evident from low-arsenic concentrations in shallow dug wells, but higher concentrations in deeper boreholes, which has been observed elsewhere in Canada highlighting the geogenic nature of arsenic contamination present in this study area (Peters et al. 1999; Bondu et al. 2017). The strong correlation between arsenic and pH is often observed in alkaline waters (Shaji et al. 2021).

**Fig. 5:**
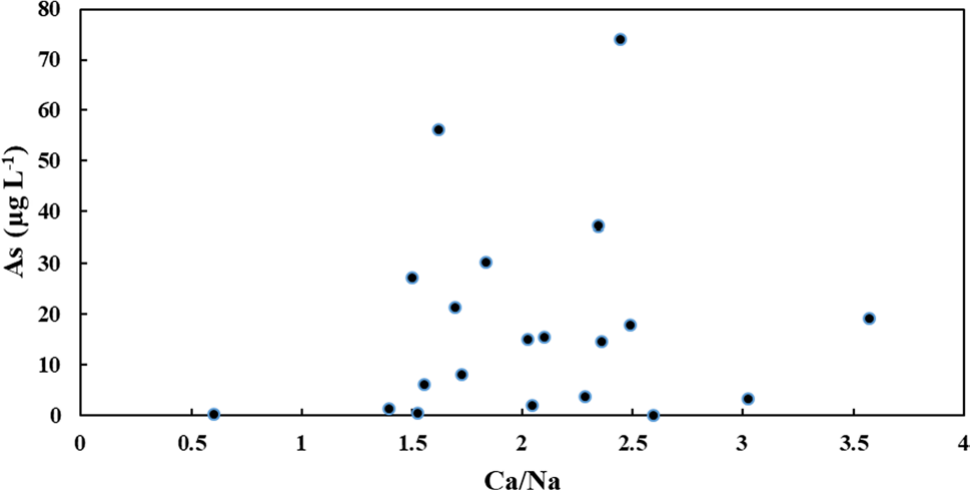
Relationship between arsenic concentrations and Ca/Na ratios in groundwaters for 2016 data

The small spatial variations in arsenic concentrations reported in the literature can be observed in this study, especially at the dwelling containing BH-61 and BH-60. At this dwelling, BH-61 was drilled in 1995 which had an arsenic concentration of 15.4 µg L^−1^ (2016). BH-60 was drilled deeper in 2007 to avoid high arsenic concentrations but had an arsenic concentration of 73.95 µg L^−1^ in 2016 (well only sampled in 2016). During sampling, the homeowner indicated that the well driller encountered quartz veins at depth**.** These wells are 27.5 m apart which illustrate that arsenic concentrations can vary over small spatial scales (Ravenscroft et al. 2009; Smedley and Kinniburgh 2013; Ayotte et al. 2017). In these fractured aquifers, this large spatial variability is mainly governed by groundwater flow through influencing the dilution process and hydrochemistry with previous studies illustrating a similar process (Peters 2008; Smedley et al. 2007; Bondu et al. 2016). Groundwater samples had low Ca/Na ratios with boreholes containing elevated arsenic concentrations having lower ratios (Fig. 5 and Fig. S5). More geochemically evolved groundwater (older) is expected to contain elevated dissolved arsenic from increased reaction time between minerals and water, i.e. low Ca/Na ratios. In comparison, less geochemically evolved groundwater (younger groundwater) contains low dissolved arsenic, i.e. higher Ca/Na ratios (Ryan et al. 2013; Bondu et al. 2016). As the geochemical signature of groundwater evolves along the flow path arsenic concentrations increase with depth (Fig. 4a) (Smedley et al. 2007; Bondu et al. 2016), Ca/Na ratios calculated in this present study are mixed which suggests a mixing of younger and older groundwaters along the flow path presumably at fracture points which is supported given that a significant portion of groundwater is recharge water (Fig S3a–b).

Good recovery from field speciation using SPE-based methodology was achieved (Table 2) with confirmatory analysis by HPLC-ICP-MS showing similar results (data not shown) with previous studies (O’Reilly et al. 2010; Watts et al. 2010). The dominant species was arsenate (As^V^) which ranged in concentration from 0.09 to 64.3 µg L^−1^.

**Table 2:** Summary of dissolved arsenic species in groundwater samples from 2016 using SPE methodology (where *n* = 20)

ID	pH	Eh	Dissolved As (µg L^−1^)	As^III^		As^V^		DMA^V^		MA^V^		Σ As Species (µg L^−1^)	Recovery (%)
				Conc. (µg L^−1^)	%	Conc. (µg L^−1^)	%	Conc. (µg L^−1^)	%	Conc. (µg L^−1^)	%		
DW-11	6.5	475.6	0.3	0.5	59.2	0.1	11.1	0.2	23.8	0.1	5.9	0.9	275.7
BH-23	7.7	460.3	19.2	0.5	1.7	25.3	94.2	1.0	3.6	0.1	0.5	26.9	140.2
BH-20	7.9	367.1	37.2	0.1	0.2	29.4	96.4	1.0	3.2	0.1	0.2	30.5	81.9
BH-21	7.4	440.9	17.9	0.1	0.6	14.5	95.6	0.5	3.5	0.04	0.3	15.3	84.7
BH-64	7.9	400.0	27.2	0.1	0.3	20.9	95.9	0.8	3.8	0.01	0.1	21.7	80.1
BH-47	7.4	441.6	0.1	0.1	21.6	0.1	34.0	0.1	39.7	0.01	4.6	0.3	232.7
BH-46	7.9	452.0	6.1	0.1	2.5	4.8	92.6	0.2	4.7	0.01	0.2	5.1	85.0
DW-17	6.8	-52.1	0.4	0.1	15.1	0.3	47.7	0.2	35.0	0.01	2.2	0.6	143.4
BH-40	8.0	-121.5	14.6	0.03	0.3	11.8	95.5	0.5	4.1	0.01	0.2	12.4	84.8
BH-29	7.9	224.9	15.0	0.1	0.8	10.9	94.8	0.5	4.3	0.01	0.1	11.5	76.4
BH-26	7.8	184.9	21.4	0.2	1.1	16.6	95.1	0.7	3.7	0.02	0.1	17.4	81.2
BH-68	7.4	262.0	1.3	0.1	4.4	0.9	73.9	0.2	20.7	0.01	1.1	1.2	88.2
BH-74	7.4	340.2	3.3	0.1	2.1	2.1	85.8	0.3	11.6	0.01	0.6	2.5	75.2
DW-25	8.1	300.7	56.1	0.4	0.8	42.4	95.7	1.5	3.5	0.02	0.0	44.3	79.1
DW-28	7.1	316.4	2.1	0.03	1.7	1.7	86.9	0.2	10.8	0.01	0.6	2.0	96.1
BH-70	8.3	302.5	8.1	0.01	0.1	6.6	94.4	0.4	5.4	0.01	0.1	7.0	85.8
BH-61	7.6	254.8	15.5	0.6	6.0	9.0	88.3	0.5	5.3	0.03	0.4	10.2	66.0
BH-60	7.5	242.5	73.9	0.04	0.1	64.3	96.5	2.3	3.4	0.02	0.0	66.6	90.1
BH-67	6.9	261.4	3.8	0.04	1.1	2.9	91.4	0.2	7.1	0.01	0.3	3.3	85.3
BH-39	7.8	296.4	30.4	0.6	2.1	25.2	94.3	0.9	3.5	0.02	0.1	26.7	88.1

**Fig. 6:**
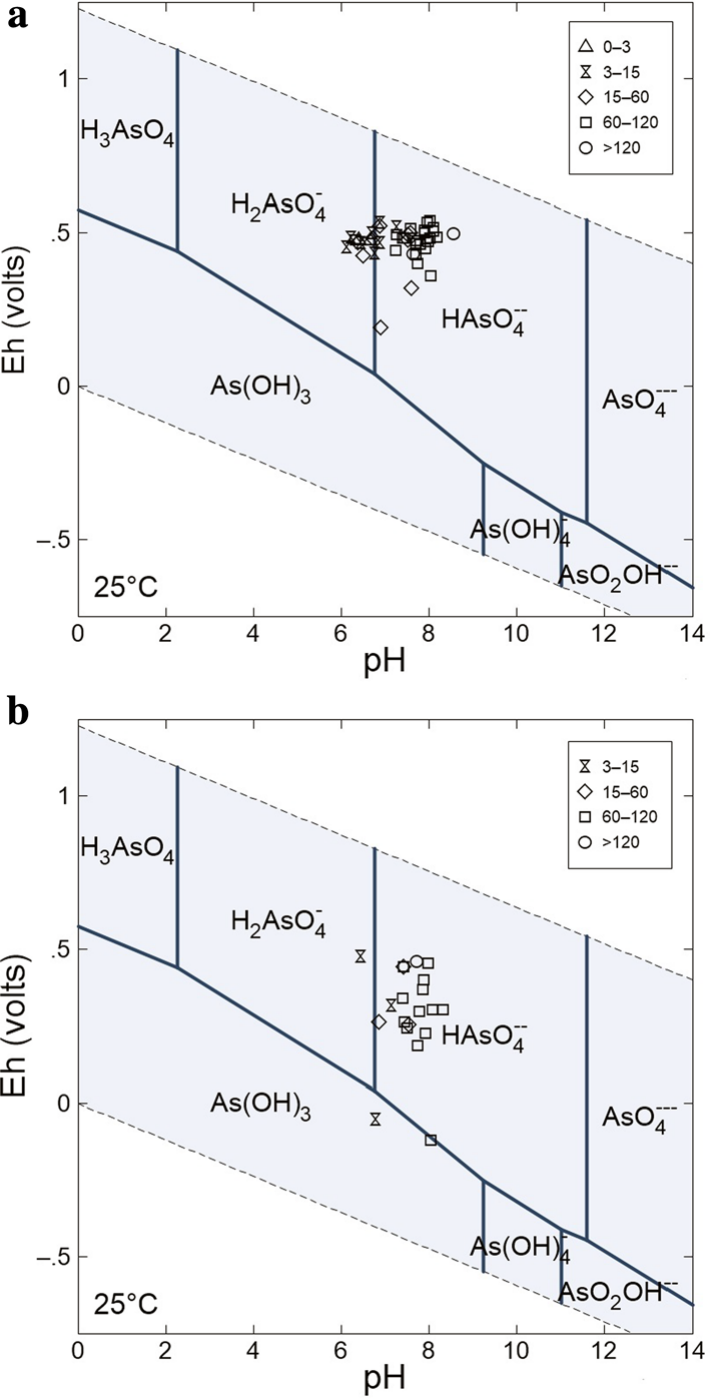
Eh–pH diagrams for **a** 2015 and **b** 2016 as a function of groundwater well depth categories (m)

The Eh–pH diagrams show that in 2015 pentavalent arsenic was in the form of HAsO_4_^−^, however in comparison, in 2016 pentavalent arsenic was in the form of H_2_AsO_4_^−^ (Fig. 6a–b). By contrasting two spatially close wells (< 5 m apart; BH-40 and DW-17) have arsenic in the form of As(OH)_3_ and are composed of a small proportion of As^III^ in 2016 (Fig. 6b); however, the Eh–pH diagrams from 2015 (Fig. 6a) show these are present as HAsO_4_^−^ and H_2_AsO_4_^−^. These data illustrate that using Eh–pH diagrams solely to infer arsenic speciation must be done with caution. It must be noted that these may be useful for informative purposes only. Both the organic methylarsenicals MA^V^ and DMA^V^ were detected in groundwaters reaching a maximum concentration of 0.12 µg L^−1^ and 2.25 µg L^−1^_,_ respectively. The presence of these methylated arsenic species is typical of microbial-medicated methylation reactions (Smedley and Kinniburgh 2013). Despite occurring in many regions of the world, these microbial methylation reactions were considered insignificant with the arsenic cycle being assumed to be limited to redox transformations between arsenite and arsenate (Oremland and Stolz 2003; Maguffin et al. 2015). Elsewhere low concentrations of methylarsenicals have been reported in natural waters resulting from microbial processes (O′Reilly et al. 2010; Christodoulidou et al. 2012; Maguffin et al. 2015; Bondu et al. 2017). Strong positive correlations were also evident with dissolved arsenic and As^V^ (*τ* = 0.93), dissolved arsenic and DMA^V^ (*τ* = 0.89), and moderate positive correlations with As^III^ and MA^v^ (*τ* = 0.49), As^V^ and DMA^V^ (*τ* = 0.91) and DMA^V^ and MA^v^ (*τ* = 0.38) (correlation coefficients from 2016 used for arsenic species data). These correlations illustrate that there is a biomethyation pathway of inorganic arsenic occurring in these natural waters (Maguffin et al. 2015). The presence of these methylated arsenicals warrants further work as recently the toxicity of these methylated species has been found to be greater than previously considered (Wang et al. 2014; Mestrot et al. 2013) and to further understand arsenic cycling and biomethylation processes within the aquifer system.

In this study, the dominant aqueous control is pH rather than Eh (which is usually reported in the literature) which is evident from the correlation of As^III^/As^V^ with pH (*τ* = 0.34) and lack of correlation with Eh (*τ* = 0.07).

#### Geochemical Controls on Groundwater Geochemistry

Thermodynamic modelling (Table S11) revealed that several Fe, Mn, Cu and Al oxides were supersaturated in groundwater including cupric ferrite, cuprous ferrite, diaspore, Fe(OH)_2_.7Cl.3, Fe(OH)_3_ (a), goethite, hematite, maghemite, magnetite, pyrophyllite, K-mica and adularia. Several minerals were undersaturated, (they could dissolve into solution) which included alunite (KAl_3_(SO_4_)_2_(OH)_6_), anorthite (CaAl_2_Si_2_O_8_), arsenolite (As_2_O_3_) and fluorite (CaF_2_) (data not shown) with specific arsenic bearing minerals including scorodite, As_2_O_5_(cr) and claudetite (As_2_O_3_). These undersaturated arsenic minerals indicated that mobilised arsenic should remain dissolved once released into solution (Sappa et al. 2014). Given the high adsorption capacity of Fe and Mn oxyhydroxides and clay minerals (K-mica and kaolinite) these phases can control the reactivity and thus the concentrations of arsenic and other oxyanion forming elements in this groundwater system (Rango et al. 2013). Several arsenic-bearing phases are undersaturated in the groundwater and have limited influence on the mobilisation of arsenic. Given that concentrations of trace elements are low in groundwaters (i.e. Fe, Mn, Al and Cu), it is more plausible that these mineral phases are controlling arsenic concentrations in the groundwater through desorption processes (Palumbo-Roe et al. 2007). Indeed, the principal pathway of controlling groundwater arsenic is through adsorption–desorption with Fe and Mn oxyhydroxides (Rango et al. 2013) with high proportions of particulate phases of Fe, Mn and Al present in these groundwaters (Table S10). Certain iron minerals (siderite and scorodite) were unsaturated and may explain the low concentrations of iron in groundwater. The poor correlation between Fe and As may result from precipitation of Fe oxyhydroxides. As mainly iron minerals were supersaturated, they can precipitate out and desorb arsenic from the surface sites of particulate iron minerals. During sampling, iron precipitates were noted in many sampling bottles (predominately on unused groundwater wells in a reducing environment) and this would further explain the poor correlation of arsenic with iron and manganese (Sracek et al. 2004).

### Multivariate Analysis

Results from the PCA analysis explained 90.9% of the variance in 10 principal components (PCs) with 25.1% and 20.2% of the variance in both PC1 and PC2, respectively (Table S12) (interpretations made using 2016 data). Overall PC1 describes general water–rock interactions as strong and moderate positive PC loadings for cations and anions are present (Fig. S6). PC2 is characterised by strong positive PC loadings for As, DMA, As^V^, Sb, moderate positive PC loadings for depth, pH, MA, Se, Mo, Pb, U and moderate negative PC loadings for Al, Mn, Fe. The inverse loadings for Al, Mn and Fe for PC2 indicate that with increasing concentrations of arsenic (and other oxyanions) in addition to increased well depth and pH values, a decrease in concentration of transition series trace elements occurred. This factor illustrates that the concentrations of oxyanions (As, Se, Mo, Sb and U) in groundwater are potentially controlled by deeper recharging groundwater at alkaline pH which promotes desorption of oxyanions from the surface of Al, Fe and Mn secondary mineral phases. While the SI indicated that Cu minerals may also have a role in desorption, the loading of Cu in this PC was weak. Furthermore, a smaller proportion of Cu was in the particulate form when compared to Fe, Mn and Al (Table S10). Other oxyanions (U, Mo, Se and F) are present as positive loadings with pH and depth with negative loadings of both Fe and Mn in PC4. This may be indicative of another source of oxyanions into groundwater with no association with arsenic.

Similar results were observed with Q-mode HCA showing 5 clusters in each sampling year (Table 3). In the first cluster depth, pH and arsenic are present with other oxyanions (Se, Mo, Sb and U). Redox parameters were associated with Fe, Cu and Mn in addition to transition metals and cations. In both years cluster 4 is associated with HCO_3_^−^, SO_4_^2−^, Ba, Mg and Sr which may influence groundwater geochemistry.

**Table 3:** HCA parameters (Q-mode)

Cluster	Parameters	
	2015	2016
1	Depth Temp pH F As Se Mo Sb W U	Depth pH F As As^V^ DMA^V^ Se Mo SB W U
2	Conductivity TDS Cl B Co Ni Ca	Temp d-O_2_ Al V Cr
3	Eh Mn Fe Cu Zn Ag Cd Pb K	Conductivity TDS Eh Cl B Zn As^III^ MA^V^ Ag Cd Sn Pb Ca Na
4	HCO_3_^−^, SO_4_^2−^ Ba Mg Sr	HCO_3_^−^, SO_4_^2−^ Ba Mg Sr
5	Be Al Ti V Cr Si Na	Ti Mn Fe Co Ni Cu Si K

**Fig. 7:**
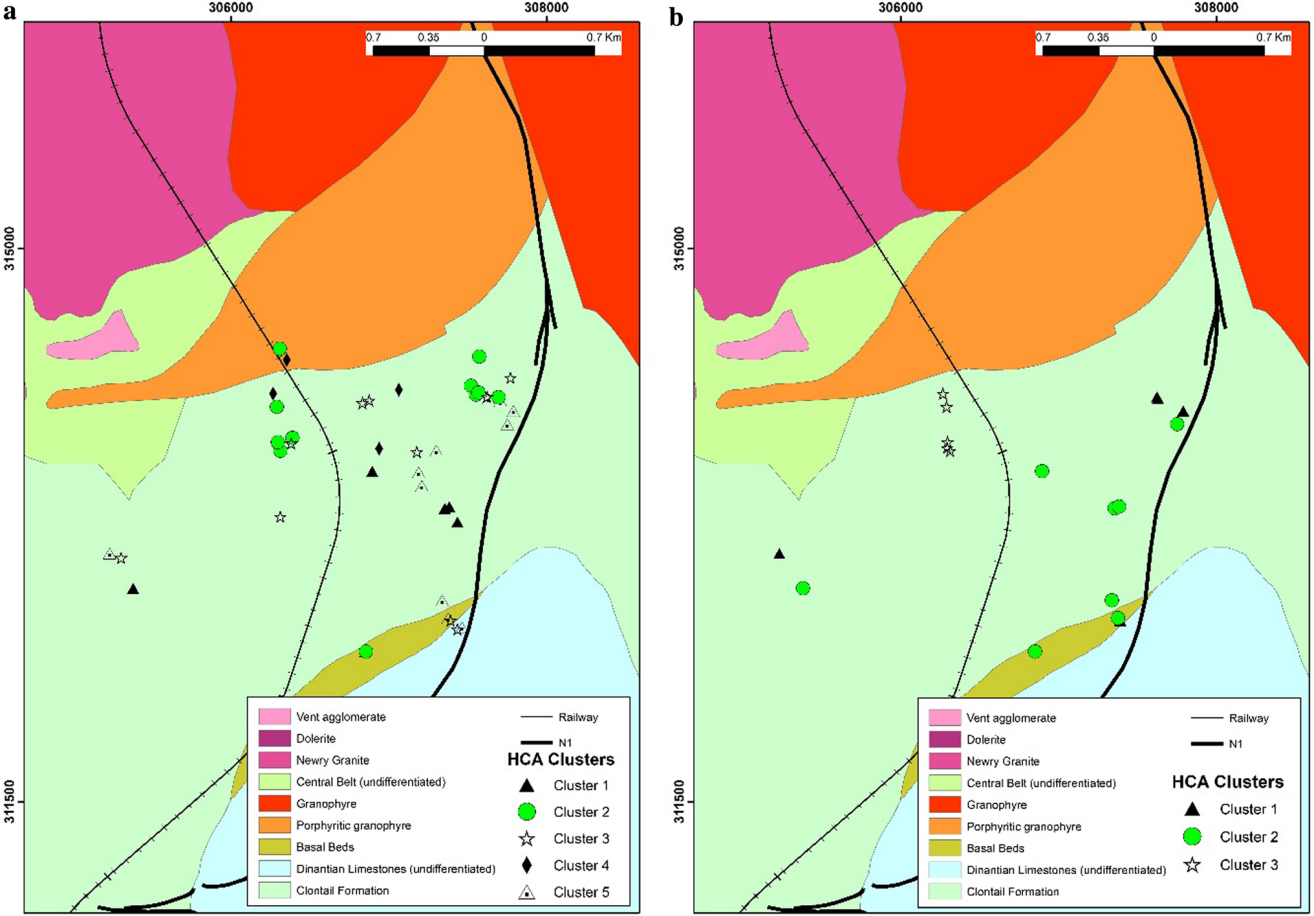
HCA clustering map for monitoring sites in **a** 2015 and **b** 2016 (R-mode) overlaid on bedrock geology (1:100 K)

Additionally, HCA using R-mode analysis showed different spatial clusters for both 2015 (5 clusters) and 2016 (3 clusters) (Fig. 7a–b). For 2015 data there were 6, 11, 10, 5 and 11 sites for Cluster1-Cluster5. There were 5, 8 and 7 sites for Cluster1-3 for 2016 data. For 2015 both Cluster 1 and Cluster 5 have strong positive loadings for depth, temperature, pH and several oxyanions which are mainly spatially clustered near each other. It is worth noting the main difference in these two clusters is that Cluster 5 has positive loadings for Ca, SO_4_^2−^ and HCO_3_^−^ indicating that these elevated concentrations of arsenic are associated with recharging Ca-HCO_3_^−^ waters. Given the dominance of Ca-HCO_3_^−^ groundwaters, the other spatial cluster is not associated with Ca or HCO_3_^−^ indicating potential mixing of groundwater from fracture points in the borehole. Similar observations are seen with Cluster 2 in 2016. In addition, SO_4_^2−^ is associated with Cluster 5. Cluster 3 of 2016 and Cluster 2 and 3 of 2015 showed associations mainly to the north of study area and had positive loadings mainly for Al, Mn, Fe and Cu. This cluster is considered a low-arsenic cluster and interestingly in 2016 d-O_2_ was associated with this cluster. These clusters associated with Fe and Mn groundwaters show the greatest spatial variation.

### Geochemical Controls on Arsenic Mobilisation

Elevated arsenic concentrations reported in this groundwater system in north-east Ireland result from a geogenic origin. While anthropogenic activity is not expected to be a major contributor of elevated arsenic in groundwater, previous work in this area has identified that some wells receive excess nutrient contamination (particularly NO_3_^−^) and microbiological contamination from inadequate wastewater treatment facilities (McGrory et al. 2020). In comparison, boreholes with elevated arsenic contained little to no microbiological contamination.

The mobilisation of arsenic in these groundwaters results through desorption at high pH under oxidising conditions though alkali desorption. In this present work, arsenic is likely being desorbed from Al hydroxides (e.g. diaspore), Fe oxides (e.g. hematite) and Fe hydroxides (e.g. goethite) as shown elsewhere (Sappa et al. 2014). A general trend was also observed in the same location in 2005 on a smaller subset of wells (McGrory et al. 2020). Al, Mn and Fe mineral phases are potential arsenic adsorbents in these groundwaters given that the major forms of these trace elements, especially Fe have a high proportion in the particulate form. Under such oxidising conditions the solubility of both Fe and Mn are low (Smedley et al. 2005) and this is observed from the low concentrations of these trace elements in this study. The strong correlation of arsenic and pH under oxidising conditions is characteristic of this geochemical process (Schreiber et al. 2021; Shaji et al. 2021). Under alkaline conditions, mineral surfaces are negatively charged which limits adsorption of arsenic oxyanions as the pH increases above 7 to 8.5 specific to the mineralogy of the aquifer (Ravenscroft et al. 2009; Bondu et al. 2016). As a result, elevated arsenic concentrations are often found above the threshold pH value of 7 – 8.5 in bedrock aquifers (e.g. Ayotte et al. 2003; Boyle et al. 1998; Rango et al. 2013; Ryan et al. 2013). Indeed, positive correlations of pH and arsenic are consistent with the weaker sorption of As^V^ to iron oxide surfaces at higher pH values (Smedley et al. 2002; Bhattacharya et al. 2006). This increase in pH promotes the desorption of other oxyanions including U, Se, Sb, Mo, B and V (Bhattacharya et al. 2006; Smedley et al. 2002, 2005). The correlation of arsenic with other oxyanions has been observed through alkali desorption processes in other oxidising aquifers (Smedley et al. 2002; Bhattacharya et al. 2006; Scanlan et al. 2009; Rango et al. 2013; Sappa et al. 2014). These adsorbed anions will interact with adsorption sites on the oxides in a competitive way to influence the extent of binding of each other (Smedley and Kinniburgh 2013). For example, HCO_3_^−^ can compete with As^V^ (Appelo et al. 2002) which may be occurring given strong correlation and association through multivariate analysis. In addition, PO_4_^3−^ can also compete with As^V^, but was not analysed as part of this study (Hongshao and Stanforth 2001). The presence of phosphate from either fertiliser or wastewater sources may alter the concentration of arsenic in groundwaters in this study. HCO_3_^−^ is the dominant anion in this high-arsenic groundwater, but concentrations are not as high as the often-reported exceedance of > 500 mg L^−1^ for this mobilisation process (Smedley and Kinniburgh 2013). While the association of HCO_3_^−^ and arsenic suggests there may be competition for adsorption sites, however, this may be an indicator of other geochemical processes which increased pH such as the dissolution of carbonates (Bhattacharya et al. 2006). Furthermore, the dominant arsenic species in these groundwaters is arsenate. The groundwater composition of reducing aquifers reported in areas of south-east Asia are generally typified by high concentrations of Fe, Mn, HCO_3_^−^, P, DOC and NH_4_ in conjunction with low concentrations of NO_3_^−^ and SO_4_^2−^ (Ravenscroft et al. 2009; Smedley and Kinniburgh 2013) and do not reflect the geochemical composition of groundwaters in this study.

### The Source of Arsenic

While alkali desorption explains the mobilisation of arsenic in groundwater in this area, it does not fully account for the source of arsenic. The correlation with arsenic and oxyanions, F and Sr are indicative of a volcanic source (Smedley et al. 2002; Bhattacharya et al. 2006; Sappa et al. 2014). Volcanic rocks such as tuffs have been present in numerous sites in relation to the generation of high arsenic waters (Masuda 2018). The weathering by-products of volcanic rock can include secondary silica, Fe/Mn oxides, Al hydroxides and clay minerals (Sappa et al. 2014). These secondary mineral phases play a critical role, and ultimately are a sink for arsenic and other oxyanions until their mobilisation through adsorption–desorption processes described above and elsewhere (Rango et al. 2013; Sappa et al. 2014).

An alternative source for the arsenic for AO waters can occur with sulphide mineralisation in veins and coating fracture surfaces (Ravenscroft et al. 2009). During sampling, it was remarked by the homeowner that quartz veins were noted during drilling of the well (BH-60, 73.95 µg L^−1^) which may explain the elevated concentration of arsenic in this well given that a well in the same location (27 m apart) had a moderate arsenic concentration (BH-61, 15.46 µg L^−1^). This prior weathering from primary sulphides can transfer arsenic from sulphides to secondary phases illustrating that secondary chemical pathways can liberate As to groundwater along fractures under alkali-oxic groundwater conditions (Nicholas et al. 2019). In this study location, previous work using two bedrock drill cores has shown that disseminated arsenic-bearing minerals with associated Co and Ni being identified within the basaltic dykes (Russel et al. 2018 and 2021). However, no correlations for arsenic, nickel and cobalt were observed in this present study. In addition, none were apparent for sulphate, with low levels detected in this location. Generally, mobilisation from sulphide sources (including mineralised areas) would yield high concentrations of sulphate (typically hundreds of mg L^−1^ or higher), acidity, high concentrations of Fe/Mn and variable arsenic speciation (Ravenscroft et al. 2009; Smedley and Kinniburgh 2013). These acid-waters would also give rise to increased concentrations of other trace elements including Ni, Pb, Zn, Cu and Cd with no correlations observed with arsenic. In this present study, the hydrochemistry of sulphide oxidation does not fully agree with the low concentrations of sulphate, iron and manganese were measured in this present study. Whilst both reductive dissolution and sulphide oxidation are oxic in nature, they differ in their pH, with alkaline and acidic waters reported, respectively.

However, it may be possible that in this study both sulphide oxidation and alkali desorption could operate simultaneously within the same geological terrain. This geochemical feature occurs elsewhere where the geology is often granitic, as with the geological setting of this study, i.e. granitoid intrusions into metasedimentary bedrock. For example, this was observed in areas of Finland, British Columbia and New England (Boyle et al. 1998; Ayotte et al. 2003; Loukola-Ruskeeniemi and Lahermo 2004; Ravenscroft et al. 2009; Schreiber et al. 2021). Given the presence of carbonate minerals, the acidity arising from sulphide oxidation can also be neutralised. However, in this study, only a moderate correlation is observed with SO_4_^2−^. In addition, given the presence of methylarsenicals in groundwater, some of the reactions giving rise to elevated arsenic are potentially microbially mediated, but the extent is not fully known. Further metagenomic studies would be needed to fully elucidate this pathway in the present study area and to gain insight into the biomethylation pathway of arsenic.

Most elevated arsenic concentrations occurred in wells intersected in the calcareous metasedimentary Clontail formation (Fig. 1a–b) (calcareous red-mica greywacke) with comparable calcareous metasedimentary aquifers giving rise to elevated arsenic in areas of New England (Peters et al. 1999; Ayotte et al. 2003). Elsewhere in Ireland, similar rock types are intersected with groundwater wells that contain elevated arsenic in isolated hotspots (with concentrations up to 242 µg L^−1^) (Morrison et al. 2016; McGrory et al. 2017). The prevalence of elevated arsenic in several fractured metasedimentary bedrock aquifers (including calcareous metasedimentary bedrock) as sources of elevated arsenic in groundwater have also been identified in the US (Ayotte et al. 2003 and 2006; Smedley et al. 2007; Ryan et al. 2013 and 2015; O'Shea et al. 2015; Andy et al. 2017; Bondu et al. 2017 and 2018). Oftentimes these fractured aquifers consist of metavolcanics and metasedimentary geology which are intruded granitoids which can be overlain by younger deposits (Bondu et al. 2016 and 2018).

It has been noted that arsenic can be particularly high in certain greywacke-shale sequences in orogenic belts (Plant et al. 1998; Pan et al. 2012). Concentrations of arsenic in greywackes are reported to be about 8 mg kg^−1^ (Wedepohl 1991). In Ireland, Silurian metasedimentary lithology has been found to contain a median arsenic concentration of 24.7 mg kg^−1^ in southwest Dublin (Glennon et al. 2014). Greywackes can also contain high concentrations of manganese (Homoncik et al. 2010).

## Conclusions

This study has investigated the presence of elevated arsenic concentrations occurring in a subset of private water supply boreholes tapping into groundwater flow systems within a fractured metasedimentary bedrock aquifer. The main conclusions of this present study are summarised below:

- The geochemistry of these natural waters show that arsenic was the most detected oxyanion forming element; however, both Sb and U were found above regulated limits to a lesser extent.
- Arsenic was not detected above regulatory limits in surface dug wells but was in deeper borehole wells illustrating that surface processes such as anthropogenic contamination are not contributing to elevated arsenic concentrations in the aquifer.
- Groundwaters with elevated arsenic are characterised as oxic-alkali, low Ca/Na ratios, low Fe and Mn, with the co-occurrence of several oxyanions (Mo, Se, Sb and U).
- Mobilisation of arsenic is through the process of alkali desorption from secondary Fe oxyhydroxides; however, microbial processes may be contributing to the arsenic biomethylation pathway in the aquifer.
- The source of arsenic may be sulphide minerals within fractures in the bedrock aquifer, with transportation of arsenic and other oxyanion forming elements facilitated mainly by secondary Fe mineral phases.
- The dominant speciation of arsenic in groundwater is arsenate, with pH controlling the speciation. The application of SPE-based methodology was used to overcome many of the limitations of implementing laboratory-based speciation of arsenic.

## Supplementary Information

Below is the link to the electronic supplementary material.

Supplementary file1 (PDF 1348 KB)

## Declarations

### Availability of Data and Material

Data generated as part of this study is available upon request from the corresponding author.

## References

[CR200] Ammann AA (2011). Arsenic speciation analysis by ion chromatography – a critical review of principles and applications. Am J Analyt Chem.

[CR1] Anderson TB (2004) Southern Uplands-Down-Longford Terrane. In: *The Geology of Northern Ireland: Our Natural Foundation*. Second edition. Geological Survey of Northern Ireland, Belfast, pp. 41–60

[CR2] Andy CM, Fahnestock MF, Lombard MA, Hayes L, Bryce JG, Ayotte JD (2017). Assessing models of arsenic occurrence in drinking water from bedrock aquifers in New Hampshire. J Contemp Water Res Educ.

[CR3] Appelo CAJ, Postma D (2005). Geochemistry, groundwater and pollution.

[CR4] Appelo CAJ, Van der Weiden MJJ, Tournasst C, Charet L (2002). Surface complexation of ferrous iron and carbonate on ferrihydrite and the mobilization of arsenic. Environ Sci Technol.

[CR6] Association of Official Analytical Chemists (AOAC) (2002) AOAC guidelines for single laboratory validation of chemical methods for dietary supplements and botanicals. AOAC International, Maryland, pp. 38

[CR7] Ayotte JD, Montgomery DL, Flanagan SM, Robinson KW (2003). Arsenic in groundwater in eastern New England: occurrence, controls, and human health implications. Environ Sci Technol.

[CR8] Ayotte JD, Nolan BT, Nuckols JR, Cantor KP, Robinson GR, Baris D, Hayes L, Karagas M, Bress W, Silverman DT, Lubin JH (2006). Modelling the probability of arsenic in groundwater in New England as a tool for exposure assessment. Environ Sci Technol.

[CR9] Ayotte JD, Medalie L, Qi SL, Backer LC, Nolan BT (2017). Estimating the high-arsenic domestic-well population in the conterminous United States. Environ Sci Technol.

[CR10] Ayotte JD, Nielsen MG, Robinson Jr GR, Moore RB (1999) Relation of arsenic, iron, and manganese in ground water to aquifer type, bedrock lithogeochemistry, and land use in the New England coastal basins. Water-Resources Investigations Report 99–4162. United States Geological Survey, New Hampshire, p. 70

[CR11] Ball JW, Nordstrom DK (1991). User’s manual for WATEQ4F, with revised thermodynamic data base and test cases for calculating speciation of major, trace, and redox elements in natural waters. United States Geological Survey (USGS) Open-File Report 91–183. USGS, Menlo Park, pp. 195

[CR12] Bartlett MS (1950). Tests of significance in factor analysis. Br J Math Stat Psychol.

[CR13] Bethke CM, Yeakel S (2018). Geochemists workbench user’s guides.

[CR14] Bhattacharya P, Claesson M, Bundschuh J, Scacek O, Fagerberg J, Jacks G, Martin RA, Storniolo AD, Thir JM (2006). Distribution and mobility of arsenic in the Rio Dulce alluvial aquifers in Santiago del Estero Province, Argentina. Sci Total Environ.

[CR15] Blake JM, Peters SC (2015). The occurrence and dominant controls on arsenic in the Newark and Gettysburg Basins. Sci Total Environ.

[CR16] Bondu R, Cloutier V, Rosa E, Benzaazoua M (2016). A review and evaluation of the impacts of climate change on geogenic arsenic in groundwater from fractured bedrock aquifers. Water Air Soil Pollut.

[CR17] Bondu R, Cloutier V, Rosa E, Benzaazoua M (2017). Mobility and speciation of geogenic arsenic in bedrock groundwater from the Canadian Shield in western Quebec, Canada. Sci Total Environ.

[CR18] Bondu R, Cloutier V, Rosa E (2018). Occurrence of geogenic contaminates in private wells from a crystalline bedrock aquifer in western Quebec, Canada: geochemical sources and health risks. J Hydrol.

[CR19] Boyle DR, Turner RJW, Hall GEM (1998). Anomalous arsenic concentrations in groundwaters of an island community, Bowen Island, British Columbia. Environ Geochem Health.

[CR20] Bräuner CV, Nordsborg RB, Andersen ZJ, Tjønneland A, Loft S, Raaschou-Nielsen O (2014). Long-term exposure to low-level arsenic in drinking water and diabetes incidence: a prospective study of the diet, cancer and health cohort. Environ Health Perspect.

[CR21] Chadha DK (1999). A proposed new diagram for geochemical classification of natural waters and interpretation of chemical data. Hydrogeol J.

[CR22] Christodoulidou M, Charalambous C, Aletrari M, Kanari PN, Petronda A, Ward NI (2012). Arsenic concentrations in groundwaters of Cyprus. J Hydrol.

[CR23] Collins JF, Daly E, Sweeney J, Walsh M, White J, Wright G (2001) Terrestrial data. In: Holden NM (Ed.) Agro-Meteorological Modelling: Principles, Data and Applications. Joint Working Group on Applied Agricultural Meteorology, Dublin, pp. 80–135

[CR24] Cooper MR, Johnston TP (2004). Late Palaeozoic Intrusives. In: Mitchell WI (Ed.) The Geology of Northern Ireland: Our Natural Foundation Second edition. Geological Survey of Northern Ireland, Belfast, pp. 61–68

[CR25] Creasey CI, Flegal AR (1999). Elemental analyses of groundwater: demonstrated advantage of low-flow sampling and trace metal clean techniques over standard techniques. Hydrogeol J.

[CR26] Cullen WR, Reimer KJ (1989). Arsenic speciation in the environment. Chem Rev.

[CR27] Daughney CJ, Baker T, Jones A, Hanson C, Davidson P, Thompson M, Reeves RR, Zemansky GM (2007). Comparison of groundwater sampling methods for State of the Environment monitoring in New Zealand. J Hydrol.

[CR28] DeSimone LA, Hamilton PA, Gilliom RJ (2009). Quality of water from domestic wells in principal aquifers of the United States, 1991–2004. National Water-Quality Assessment Program Circular 1332. United States Geological Survey, Reston, pp. 58

[CR29] Dial AR, Misra S, Landing WM (2015). Determination of low concentrations of iron, arsenic, selenium, and other trace elements in natural samples using an octupole collision/reaction cell equipped with quadrupole-inductively coupled plasma mass spectrometer. Rapid Commun Mass Spectrom.

[CR30] Drummer TJB, Yu ZM, Nauta L, Murimboh JD, Parker L (2015). Geostatistical modelling of arsenic in drinking water wells and related toenail arsenic concentrations across Nova Scotia, Canada. Sci Total Environ.

[CR31] Environmental Protection Agency (EPA) (2013) Borehole construction and wellhead protection. Advance Note No. 14. Environmental Protection Agency, Wexford, pp. 36

[CR32] Erickson ML, Yager RM, Kauffman LJ, Wilson JT (2019). Drinking water quality in the glacial aquifer system, northern USA. Sci Total Environ.

[CR33] Fitzgerald WF, Davidson CI (1999). Clean hands, dirty hands: clair patterson and the aquatic biogeochemistry of mercury. Clean Hands: Clair Patternson's Crusade against Environmental Lead Contamination.

[CR34] Garelick H, Jones H, Dybowska A, Valsami-Jones E (2009). Arsenic pollution sources. Rev Environ Contam.

[CR35] Geraghty M (1997). Geology of Monaghan—Carlingford: A Geological Description to accompany the bedrock geology 1:100,000 scale map series, sheet 8/9, Monaghan–Carlingford. Geological Survey of Ireland, Dublin, pp. 60

[CR36] Gibbs RJ (1970). Mechanisms controlling world water chemistry. Science.

[CR37] Gilligan M, Costanzo A, Feely M, Rollinson GK, Timmins E, Henry T, Morrison L (2016). Mapping arsenopyrite alteration in a quartz vein-hosted gold deposit using micro beam analytical techniques. Mineral Mag.

[CR38] Glennon MM, Harris P, Ottesen RT, Scanlon RP, O'Connor PJ (2014). The Dublin SURGE project: geochemical baseline for heavy metals in topsoils and spatial correlation with historical industry in Dublin, Ireland. Environ Geochem Health.

[CR39] He S, Li P (2020). A MATLAB based graphical user interface (GUI) for quickly producing widely used hydrogeochemical diagrams. Geochemistry.

[CR40] He X, Li P, Ji Y, Wang Y, Su Z, Elumalai V (2020). Groundwater arsenic and fluoride and associated arsenicosis and fluorosis in China: occurrence, distribution and management. Expo Health.

[CR41] He X, Li P, Wu J, Wei M, Ren X, Wang D (2021). Poor groundwater quality and high potential health risks in the Datong Basin, northern China: research from published data. Environ Geochem Health.

[CR42] Hearth I, Vithanage M, Bundschuh J, Maity JP, Bhattacharya P (2016). Natural arsenic in global groundwaters: distribution and geochemical triggers for mobilization. Curr Pollution Rep.

[CR43] Helsel D (2012). Statistics for censored environmental data using minitab and R.

[CR44] Helsel DR, Hirsch RM (2002). Statistical Methods in Water Resources. Techniques of Water Resource Investigations, Book 4, Chapter A3. United States Geological Survey, pp. 522

[CR45] Hem JD (1985) Study and interpretation of the chemical characteristics of natural water. 3rd Edition. U.S. Geological Survey Water-Supply Paper 2254. USGS, Alexandria, 1–263

[CR46] Homoncik SC, MacDoland AM, Heal KV, Ó Dochartaigh BÉ, Ngwenya BT (2010). Manganese concentrations in Scottish groundwater. Sci Total Environ.

[CR47] Hongshao Z, Stanforth R (2001). Competitive adsorption of phosphate and arsenate on goethite. Environ Sci Technol.

[CR48] Hubert E, Wolkersdofer C (2015). Establishing a conversion factor between electrical conductivity and total dissolved solids in South African mine waters. Water SA.

[CR49] Institute of Geologists Ireland (IGI) (2007) Guidelines on water well construction. IGI, Dublin, pp. 24

[CR5] International Agency for Research on Cancer (IARC) (2012) Arsenic, metals, fibres and dusts. IARC Monographs on the Evaluation of Carcinogenic Risks to Humans, No. 100C. IARC Working Group on the Evaluation of Carcinogenic Risks to Humans (2009; Lyon, France). IARC, France, pp. 526 PMC4781271 23189751

[CR50] Johnson CD, Nandi A, Joyner TA, Luffman I (2017). Iron and manganese in groundwater: using kriging and GIS to locate high concentrations in Buncombe County, North Carolina. Ground Water.

[CR51] Juen LL, Aris AZ, Shan NT, Yusoff FM, Hashim Z (2015). Geochemical modelling of element species in selected tropical estuaries and coastal water of the Strait of Malacca. Procedia Environ Sci.

[CR52] Kaiser HF, Rice J (1974) Little Jiffy, mark IV. Educ Psychol Meas 34, 111–117. 10.1177%2F001316447403400115

[CR53] Kaiser HF (1960). The application of electronic computers to factor analysis. Educ Psychol Meas 20, 141–151. 10.1177%2F001316446002000116

[CR54] Kemp AES (1987). Tectonic development of the Southern Belt of the Southern Uplands accretionary complex. J Geol Soc London.

[CR55] Khamis H (2008). Measures of association. How to choose? J Diagn Med Sonogr 24, 155–162. 10.1177%2F8756479308317006

[CR56] Kim M-J, Nriagu J, Haack S (2003). Arsenic behaviour in newly drilled wells. Chemosphere.

[CR57] Kumar AR, Riyazuddin P (2010). Preservation of inorganic arsenic species in environmental water samples for reliable speciation analysis. Trends Analyt Chem.

[CR58] Leybourne MI, Johannesson KH, Asfaw A (2014). Measuring arsenic speciation in environmental media: sampling, preservation, and analysis. Rev Mineral Geochem.

[CR59] Li P, Zhang Y, Yang N, Jing L, Yu P (2016). Major ion chemistry and quality assessment of groundwater in and around a mountainous tourist town of China. Expo Health.

[CR60] Li P, Wu J, Tian R, He S, He X, Xue C, Zhang K (2018). Geochemistry, hydraulic connectivity and quality appraisal of multilayered groundwater in the Hongdunzi Coal Mine, northwest China. Mine Water Environ.

[CR61] Li P, Tian R, Liu R (2019). Solute geochemistry and multivariate analysis of water quality in the Guohua Phosphorite Mine, Guizhou Province, China. Expo Health.

[CR62] Lord G, Kim K, Ward NI (2012). Arsenic speciation of geothermal waters in New Zealand. J Environl Monitor.

[CR63] Loukola-Ruskeeniemi K, Lahermo P (2004). Arsenic in Finland: distribution, environmental impacts and risks. Geological Survey of Finland, Helsinki, pp. 176.

[CR64] Lu P, Zhu C (2011). Arsenic Eh-pH diagrams at 25°C and 1 bar. Environ Earth Sci.

[CR65] Lusty PAJ, Scheib C, Gunn AG, Walker ASD (2012). Reconnaissance-scale prospectively analysis for gold mineralisation in the Southern Uplands-Down-Longford Terrane, Northern Ireland. Nat Resour Res.

[CR201] Maguffin SC, Kirk MF, Daigle AR, Hinkle SR, Jin Q (2015). Substantial contribution of biomethylation to aquifer arsenic cycling. Nat Geosci.

[CR66] Martínez-Bravo Y, Roig-Navarro AF, Lópex FJ, Hernádez F (2001). Multielemental determination of arsenic, selenium and chromium(VI) species in water by high-performance liquid chromatography-inductively coupled plasma mass spectrometry. J Chromatogr A.

[CR67] Masuda H (2018). Arsenic cycling in the Earth’s crust and hydrosphere: interaction between naturally occurring arsenic and human activities. Prog Earth Planet Sci.

[CR68] May TW, Wiedmeyer RH (1998). A table of polyatomic interferences in ICP-MS. At Spectrosc.

[CR202] McCleskey RB, Nordstorm DK, Maest AS (2004). Preservation of water samples for arsenic (III/V) determinations: an evaluation of the literature and new analytical results. Appl Geochem.

[CR69] McConnell B, Philcox M, Geraghty, M (2001). Geology of Meath: a geological description to accompany the bedrock geology 1:100,000 scale map series, sheet 13, Meath. Dublin, Geological Survey of Ireland, pp. 7

[CR73] McGrory E (2020) Environmental aqueous geochemistry of arsenic in groundwater: occurrence, speciation and biogeochemical processes. Unpublished PhD Dissertation. National University of Ireland, Galway, Galway, Ireland

[CR70] McGrory ER, Brown C, Bargary N, Hunter Williams N, Mannix A, Zhang C, Henry T, Daly E, Nicholas S, Petrunic BM, Lee M, Morrison L (2017). Arsenic contamination of drinking water in Ireland: a spatial analysis of occurrence and potential risk. Sci Total Environ.

[CR71] McGrory E, Holian E, Alvarez-Iglesias A, Bargary N, McGillicuddy EJ, Henry T, Daly E, Morrison L (2018). Arsenic in groundwater in south west Ireland: occurrence, controls, and hydrochemistry. Front Environ Sci.

[CR72] McGrory E, Holian E, Morrison L (2020). Assessment of groundwater processes using censored data analysis incorporating non-detect chemical, physical, and biological data. J Contam Hydrol.

[CR74] McKinley JM, Grunsky E, Mueller U (2017). Environmental monitoring and peat assessment using multivariate analysis of regional-scale geothermal data. Math Geosci.

[CR75] McMahon PB, Chapelle FH (2008). Redox processes and water quality of selected principal aquifer systems. Ground Water.

[CR76] McNeil VH, Cox ME (2000). Relationship between conductivity and analysed composition in a large set of natural surface-water samples, Queensland, Australia. Environ Geol.

[CR77] Meade F, Troll V, Chew D, Meighan I, Cooper M, Emeleus H (2010). Caldera controversy at the Ring of Cullion, County Down. Earth Sci Ireland Mag.

[CR78] Mestrot A, Planer-Friedrich B, Feldmann J (2013). Biovalatilisaion: a poorly studied pathway of the arsenic biogeochemical cycle. Environ Sci Process Impacts.

[CR79] Michalke B (2003). Element speciation definitions, analytical methodology, and some examples. Ecotox Environ Safe.

[CR80] Montcoudiol N, Molson J, Lemieux J-M (2014). Groundwater geochemistry of the Outaouais Regions (Québec, Canada): a regional-scale study. Hydrogeol J.

[CR81] Moon K, Guallar E, Navas-Acien A (2012). Arsenic exposure and cardiovascular disease: an updated systematic review. Curr Atheroscler Rep.

[CR82] Morris JH, Steed GM, Wilbur DG, Crone RWS, Finlay S, Pennell W, Pyne JF (1986). The Lisglassan-Tullybuck deposit, County Monaghan: Sb-As-Au vein mineralization in Lower Palaeozoic greywackes. The Geology and Genesis of Mineral Deposits in Ireland (Andrew CJ.

[CR83] Morrison L, McGrory E, Brown C (2016). National assessment of arsenic within groundwater: a case study with Ireland. In: Proceedings of the Sixth International Congress on Arsenic in the Environment (As 2016) (Bhattacharya P, Vahter M, Jarsjö J, Kumpiene J, Ahmad A, Sparrenbom C, Jacks G, Donselaar ME, Bundschuh J, Naidu R, Eds). CRC Press, Boca Raton, pp. 33–34

[CR84] Murcott S (2012). Arsenic contamination in the world: an international sourcebook.

[CR85] Naujokas MF, Anderson B, Ahsan H, Aposhian HV, Graziano JH, Thompson C, Suk WA (2013). The broad scope of health effects from chronic arsenic exposure: update on a worldwide public health problem. Environ Health Perspect.

[CR86] Nicholas SL, Russell A, McDermott FP, McGrory E, Morrison L (2019). XAS measurements of solid-phase arsenic speciation in a fractured bedrock aquifer. AGU Fall Meeting Abstracts.

[CR87] Nordstrom DK (1977). Thermochemical redox equilibria of ZoBell’s solution. Geochim Cosmochim Acta.

[CR88] O’Reilly J, Watts MJ, Shaw RA, Marcilla AL, Ward NI (2010). Arsenic contamination of natural water in San Juan and La Pampa, Argentina. Environ Geochem Health.

[CR89] Oremland RS, Stolz JF (2003). The ecology of arsenic. Science.

[CR90] O'Shea B, Stransky M, Leitheiser S, Brock P, Marvinney RG, Zheng Y (2015). Heterogeneous arsenic enrichment in meta-sedimentary rocks in central Maine, United States. Sci Total Environ.

[CR91] Palumbo-Roe B, Klinck B, Cave M, Mukherjee AB, Bundschuh J, Zevenhoven R, Loeppert RH (2007). Arsenic speciation and mobility in mine wastes from a copper-arsenic mine in Devon, UK: a SEM, XAS, sequential chemical extraction study. Arsenic in Soil and Groundwater Environment (Bhattacharya P.

[CR92] Pan J, Chon H-S, Cave MR, Oates CJ, Plant JA, Voulvoulis N, Ragnarsdottir KV (2012). Toxic trace elements. Pollutants, human health and the environment: a risk based approach (Plant JA.

[CR93] Peters SC (2008). Arsenic in groundwaters in the Northern Appalachian Mountain belt: a review of patterns and processes. J Contam Hydrol.

[CR94] Peters SC, Blum JD, Klaue B, Karagas MR (1999). Arsenic occurrence in New Hampshire drinking water. Environ Sci Technol.

[CR95] Plant JA, Gunn AG, Rollin KE, Stone P, Morrissey CJ, Norton GE, Simpson PR (1998). The MIDAS project (multiple data-set analysis for gold in Europe): evidence from the British Caledonides. T I Min Metall.

[CR96] Plant JA, Kinniburgh DG, Smedley PL, Fordyce FM, Klinck BA, TurekianHolland KKHD (2003). Arsenic and selenium. Volume 9 Treatise on Geochemistry.

[CR97] Puls RW, Clark DA, Bledsoe B (1992). Metals in ground water: sampling artifacts and reproducibility. Hazard Waste Hazard.

[CR98] Rango T, Vengosh A, Dwyer G, Bianchini G (2013). Mobilization of arsenic and other naturally occurring contaminants in groundwater of the Main Ethiopian Rift aquifers. Water Res.

[CR203] Ravenscroft P, Brammer H, Richards K (2009). Arsenic pollution: a global synthesis.

[CR99] Ren X, Li P, He X, Su F, Elumalai V (2021). Hydrogeochemical processes affecting groundwater chemistry in the central part of the Guanzhong Basin, China. Arch Environ Contam Toxicol.

[CR100] Reyes FAP, Crosta GB, Frattini P, Basirico S, Della Pergola R (2015). Hydrogeochemical overview and natural arsenic occurrence in groundwater from alpine springs (upper Valtellina, Northern Italy). J Hydrol.

[CR101] Robins NS, Misstear BDR (2000). Groundwater in the Celtic regions. Geol Soc Spec Publ.

[CR102] Russel A, McDermott F, Henry T, Morrison L (2018). Arsenic contamination of groundwater in Ireland; occurrences and sources. Geophys Res Abstr.

[CR103] Russel A, McDermott F, McGrory E, Cooper M, Henry T, Morrison L (2021). As-Co-Ni sulfarsenides in Palaeogene basaltic cone sheets as sources of groundwater arsenic contamination in Co. Louth, Ireland. Appl Geochem 10.1016/j.apgeochem.2021.104914

[CR104] Rust BR (1965). The stratigraphy and structure of the Whithorn area of Wigtownshire, Scotland. Scott J Geol.

[CR105] Ryan PC, Kim J, Wall AJ, Moen JC, Corenthal LG, Chow DR, Sullivan CM, Bright KS (2011). Ultramafic-derived arsenic in a fractured bedrock aquifer. Appl Geochem.

[CR106] Ryan PC, Kim JJ, Mango H, Hattori K, Thompson A (2013). Arsenic in fractured bedrock slate aquifer system, New England, USA: influence of bedrock geochemistry, groundwater flow paths, redox and ion exchange. Appl Geochem.

[CR107] Ryan PC, West DP, Hattori K, Studwell S, Allen DN, Kim J (2015). The influence of metamorphic grade on arsenic in metasedimentary bedrock aquifers: a case study from western New England, USA. Sci Total Environ.

[CR108] Sappa G, Ergul S, Ferranti F (2014). Geochemical modelling and multivariate evaluation of trace elements in arsenic contaminated groundwater systems of Viterbo area, (Central Italy). Springerplus.

[CR109] Scanlan BR, Nicot JP, Reedy RC, Kurtzman D, Mukherjee A, Nordstrom DK (2009). Elevated naturally occurring arsenic in a semiarid oxidizing system, Southern High Plains aquifer, Texas, USA. Appl Geochem.

[CR110] Schreiber ME, Mukherjee A, Scanlon BR, Aureli A, Langan S, Huo H, McKenzie AA (2021). Arsenic in groundwater in the United States: research highlights since 2000, current concerns and next steps. Global Groundwater—Source, Scarcity, Sustainability, Security, and Solutions.

[CR111] Selvakumar S, Ramkumar K, Chandrasekar N, Magesh NS, Kaliraj S (2017). Groundwater quality and its suitability for drinking and irrigational use in the Southern Tiruchirappalli district, Tamil Nadu, India. Appl Water Sci.

[CR112] Shaji E, Santosh M, Sarath KV, Prakash P, Deepchand V, Divya BV (2021). Arsenic contamination of groundwater: a global synopsis with focus on the Indian Peninsula. Geosci Front.

[CR113] Smedley PL, Kinniburgh DG, Selinus O, Alloway B, Centeno J, Finkelman RB, Fuge R, Lindh U, Smedley P (2013). Arsenic in groundwater and the environment. Essentials of Medical Geology.

[CR204] Smedley PL, Nicolli HB, Macdonald DMJ, Barros AJ, Tullio JO (2002). Hydrogeochemistry of arsenic and otherinorganic constituents in groundwaters from La Pampa, Argentina. Appl Geochem.

[CR114] Smedley PL, Kinniburgh DG, Macdonald DMJ, Nicolli HB, Barros AJ, Tullio JO, Pearce JM, Alonso MS (2005). Arsenic associations in sediments from the loess aquifer of La Pampa, Argentina. Appl Geochem.

[CR115] Smedley PL, Knudsen J, Maiga D (2007). Arsenic in groundwater from mineralised Proterozoic basement rocks in Burkina Faso. Appl Geochem.

[CR116] Sracek O, Bhattacharya P, Jacks G, Gustagsson J-P, von Brömssen M (2004). Behaviour of arsenic and geochemical modelling of arsenic enrichment in aqueous environments. Appl Geochem.

[CR117] Stea F, Bianchi F, Cori L, Sicari R (2014). Cardiovascular effects of arsenic: clinical and epidemiological findings. Environ Sci Pollut Res.

[CR118] Steed GM, Morris JH (1986). Gold mineralisation in Ordovician greywackes at Clontibret, Ireland. Geol Soc Am Spec Pap.

[CR119] Stetson SJ, Erickson ML, Brenner J, Berquist EC, Kanagy C, Whitcomb S, Lawrence C (2021). Stability of inorganic methylated arsenic species in laboratory standards, surface water and groundwater under three different preservation regimes. Appl Geochem.

[CR121] Talib MA, Tang Z, Shahab A, Siddique J, Faheem M, Fatima M (2019). Hydrogeochemical characterization and suitability assessment of groundwater: a case study in Central Sindh, Pakistan. Int J Environ Res Public Health.

[CR122] Thomas MA (2007). The association of arsenic with redox conditions, depth, and ground-water age in the glacial aquifer system of the northern United States. United States Geological Survey Scientific Investigations Report 2007–5036. USGS, Reston, pp. 26

[CR123] Troll VR, Meade FC, Chew DM, Emeleus CH (2008). A new exposure of a caldera fault segment at the Slieve Gullion igneous centre: implications for the emplacement of the Early Ring-Complex. Ir J Earth Sci.

[CR124] Tsuji JS, Perez V, Garry MR, Alexander DD (2014). Association of low-level arsenic exposure in drinking water with cardiovascular disease: a systematic review and risk assessment. Toxicology.

[CR125] Ujević M, Casiot C, Duić Ž, Santo V, Dadić Ž, Sipos L, Vitale M, Gallios GP, Ivanicová L, Václaviková M (2010). Distribution and speciation of arsenic and tap waters of Eastern Croatia. Water treatment technologies for the removal of high-toxicity pollutants.

[CR126] Ullrich MK, Misiari V, Planer-Friedrich B (2016). A new method for thioarsenate preservation in iron-rich waters by solid phase extraction. Water Res.

[CR205] van Halem D, Bakker SA, Amy GL, van Dijk JC (2009). Arsenic in drinking water: a worldwide water quality concern for water supply companies. Drink Water Eng Sci.

[CR127] Vaughan APM, Johnston JD (1992). Structural constraints on closure geometry across the Iapetus suture in eastern Ireland. J Geol Soc London.

[CR128] Vaughan APM (1991) The Lowe Palaeozoic geology of the Iapetus Suture Zone in eastern Ireland. Unpublished PhD thesis, University of Dublin

[CR129] Wang P, Sun G, Jia T, Meharg AA, Zhu Y (2014). A review on completing arsenic biogeochemical cycle: microbial volatilization of arsines in environment. J Environ Sci (china).

[CR130] Wangkahad B, Mongkolsuk S, Sirikanchana K (2017). Integrated multivariate analysis with nondetects for the development of human sewage source-tracking tools using Bacteriophages of Enterococcus faecalis. Environ Sci Technol.

[CR131] Watts MJ, Button M, Brewer TS, Jenkin GRT, Harrington CF (2008). Quantitative arsenic speciation in two species of earthworms from a former mine site. J Environ Monit.

[CR132] Watts MJ, O’Reilly J, Marcilla AL, Shaw RA, Ward NI (2010). Field based speciation of arsenic in UK and Argentinean water samples. Environ Geochem Health.

[CR133] Watts MJ, O’Reilly J, Smiles CA (2007) Measurement of Arsenic Compounds in Water by HPLC-ICP-MS. British Geological Survey Open Report OR/07/021. British Geological Survey, Keyworth, pp. 27

[CR134] Wedepohl KH, Clarkson TW, Fishbein L, Mallinckrodt MG, Piscator M, Schlipköter HW, Stoeppler M, Stumm W, Sunderman FWE, Merian (1991). The composition of the upper earth’s crust and the natural cycles of selected metals. Metals and their compounds in the environment: occurrence, analysis, and biological relevance.

[CR135] Wei M, Wu J, Li W, Zhang Q, Su F, Wang Y (2021). Groundwater geochemistry and its impacts on groundwater arsenic enrichment, variation, and health risks in Yongning County Yinchuan Plain of northwest China. Expo Health.

[CR136] Weight WD (2008). Hydrogeology Field Manual.

[CR137] Wu J, Li P, Wang D, Ren X, Wei M (2020). Statistical and multivariate statistical techniques to trace the sources and affecting factors of groundwater pollution in a rapidly growing city on the Chinese Loess Plateau. Hum Ecol Risk Assess.

[CR138] Zecchin S, Crognale S, Zaccheo P, Fazi S, Amalfitano S, Casentini B, Callegari M, Zanchi R, Sacchi GA, Rossetti S, Cavalca L (2021). Adaptation of microbial communities to environmental arsenic and selection of arsenite-oxidizing bacteria from contaminated groundwaters. Front Microbiol.

[CR139] Zheng Y (2017). Lessons learned from arsenic mitigation among private well households. Curr Environ Health Rep.

