# Supplementary material for: Occurrence, Geochemistry and Speciation of Elevated Arsenic Concentrations in a Fractured Bedrock Aquifer System
Source: Arch Environ Contam Toxicol. 2021 Sep 14;81(3):414–37. doi: 10.1007/s00244-021-00887-3 (PMC8478764; doi:10.1007/s00244-021-00887-3)
Supplement: Supplementary file 1 — Supplementary file1 (PDF 1348 KB) [file 244_2021_887_MOESM1_ESM.pdf]

Supplementary Information

Occurrence, geochemistry and speciation of elevated arsenic concentrations in a fractured bedrock aquifer system

Ellen McGrory, Peter Conroy, Tiernan Henry and Liam Morrison*

Earth and Ocean Sciences, School of Natural Sciences and Ryan Institute: Environmental, Marine and Energy Research, National University of Ireland, Galway, Ireland

* Corresponding author at: Earth and Ocean Sciences, School of Natural Sciences and Ryan Institute: Environmental, Marine and Energy Research, National University of Ireland, University Road, Galway, Ireland. Tel: +00353091493200; fax +00353091525005.

E-mail address: liam.morrison@nuigalway.ie (L. Morrison)

Note 1 – Reconnaissance hydrogeological survey

In order to determine potential hot spots of metal contamination and groundwater flow patterns for further study, an initial reconnaissance hydrogeological survey was undertaken in May and June 2014 with boreholes (BH), springs (SP) and shallow groundwater sources i.e. dug wells (DW), sampled (n=107) (Fig. S1). For each sampling site, a hydrogeological questionnaire was completed with the homeowner and a screening sample was obtained (where appropriate) from either an unfiltered tap location, or from the well source. The tap was run for several minutes where the 50 cm^3 *Digi*TUBE (SCP Science, USA) was rinsed several times with water, filled and acidified to pH <2 (*Optima* HNO_3, Fisher Scientific, Dublin) and packed (Bryson Packaging™ Minigrip™ PE Bags, Fisher Scientific, Ireland). Samples were stored at 4°C until returned to the laboratory. To ensure trace element clean sampling conditions, field blank samples of Milli-Q were taken.

GPS coordinates were measured at the top of the well using a Trimble XRS Pathfinder Pro differential global positioning (DGPS) system using VRS (virtual reference station) and corrected to the nearest active global navigation satellite system (GNSS) station (Markethill, OSi) by using the appropriate RINEX files on the day of sampling for post-processing. Recording time was 20 minutes. Septic tanks at each location were also recorded using a hand-held unit (Garmin GPSMAP 62 Handheld GPS, Ireland). Summary statistics were presented in Tables S1-S3 for boreholes (BHs, n=75), dug wells (DWs, n=29) and springs (SP, n=3). Due to lack of spring sources, these were not further sampled in 2015 and 2016.

These data were not used in the statistical analysis of the paper as they represent total metal concentrations (i.e., unfiltered data), while data collected in both 2015 and 2016 contain both total and dissolved (i.e., filtered data) metal concentrations. Dissolved data were used for further interpretations.

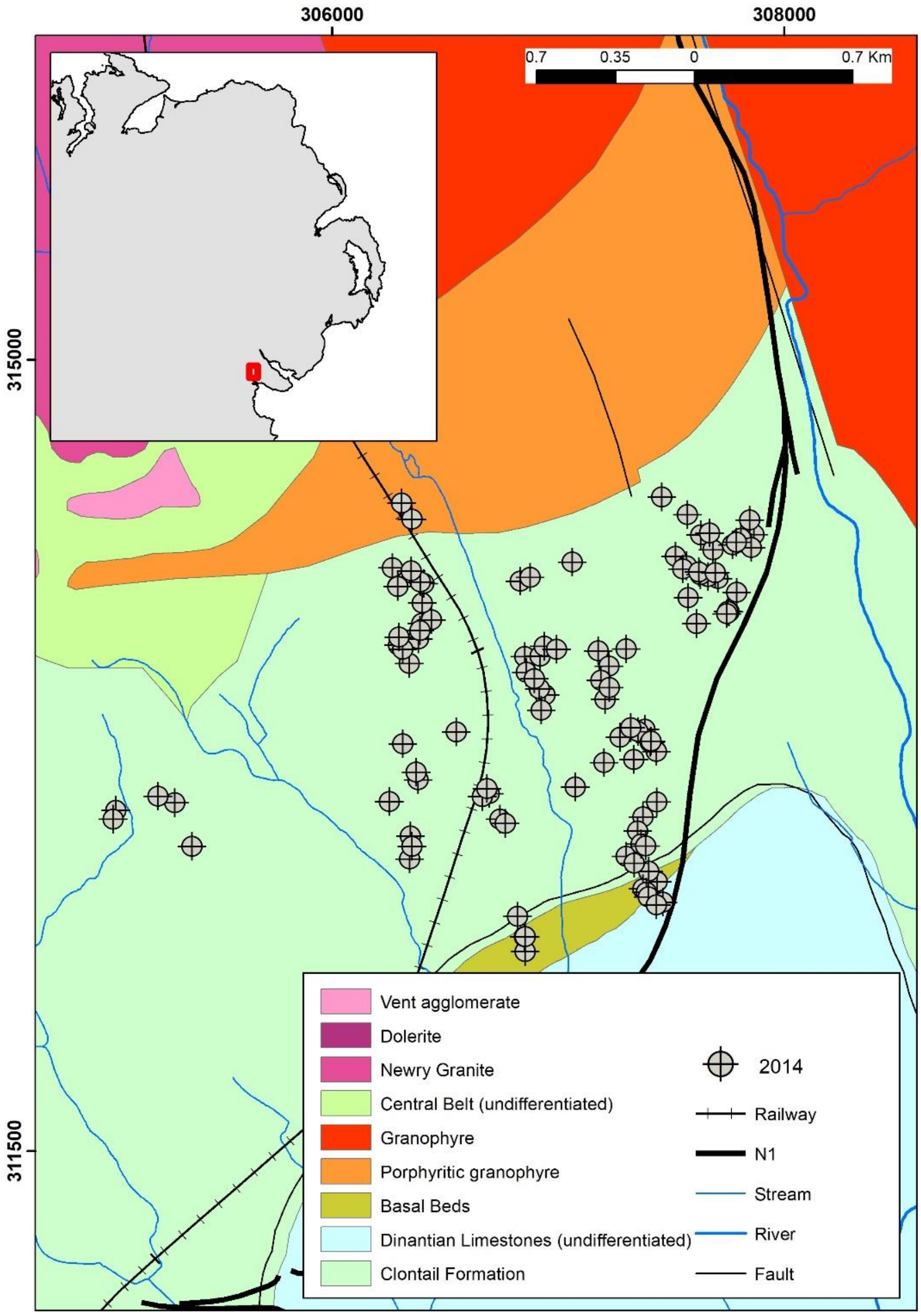

Fig. S1. Spatial distribution of groundwater sampling points collected during 2014 reconnaissance hydrogeological survey (n=107) overlaid on bedrock geology (1:100K).

Table S1. Statistical summary of hydrochemistry data on bedrock boreholes sampled in 2014 (n=75) (Limit values taken from the following references: interim guideline values (EPA 2003), drinking water regulations (EC 2014), and groundwater regulations (EC 2010).

Variable	Mean	SD	Min	Q1	Median	Q3	Max	Cen (%)	Limit	% > Limit
Depth (m)	68.8	36.1	4.1	42.8	70.0	91.6	185.0	0	-	NA
Be (μg L^{-1})	0.1	0.1	0.0	0.0	0.0	0.1	0.7	86.6	-	NA
B (μg L^{-1})	18.2	10.4	7.1	11.5	14.2	21.0	55.4	0	1000	0
Al (μg L^{-1})	96.0	447.7	1.0	1.7	4.5	48.2	3855.5	0	200	9.33
Ti (μg L^{-1})	31.5	22.1	0.3	19.1	26.6	38.1	139.1	0	-	NA
V (μg L^{-1})	2.4	5.8	0.1	0.3	0.8	1.7	42.8	0	-	NA
Cr (μg L^{-1})	0.7	1.9	0.0	0.2	0.4	0.5	14.4	2.6	50	0
Mn (μg L^{-1})	39.5	136.5	0.0	0.2	0.6	2.7	840.3	10.6	50	9.3
Fe (μg L^{-1})	2232.0	13230.0	2.0	10.0	18.0	70.0	112397.0	0	200	16
Co (μg L^{-1}	0.2	0.4	0.0	0.0	0.1	0.1	2.6	44.0	-	NA
Ni (μg L^{-1}	2.0	1.6	0.5	1.2	1.7	2.3	10.0	4.0	20	0
Cu (μg L^{-1}	31.9	52.2	0.5	6.9	15.6	35.2	379.6	0	2000	0
Zn (μg L^{-1})	112.8	192.4	3.0	13.2	30.8	118.6	879.3	0	5000	0
**As (μg L^{-1}**	**9.1**	**13.1**	**0.1**	**0.9**	**4.7**	**11.6**	**77.7**	**0**	**10**	**34.6**
Se (μg L^{-1})	0.8	1.3	0.1	0.4	0.5	0.7	9.5	0	10	0
Nb (μg L^{-1})	-	-	-	-	-	-	0.1	98.6	-	NA
Mo (μg L^{-1})	1.4	1.9	0.0	0.2	0.7	1.8	10.1	17.3	-	NA
Ag (μg L^{-1})	-	-	-	-	-	-	0.2	97.3	-	NA
Cd (μg L^{-1})	0.0	0.0	0.0	0.0	0.0	0.0	0.3	78.6	5	0
Sb (μg L^{-1})	0.3	0.5	0.0	0.1	0.1	0.3	3.3	37.3	5	0
Sn (μg L^{-1})	-	-	-	-	-	-	1.4	88.0	-	NA
Ba (μg L^{-1})	39.9	67.0	0.0	1.0	6.8	51.8	330.2	2.6	500	0
W (μg L^{-1})	-	-	-	-	-	-	10.3	86.6	-	NA
Pb (μg L^{-1})	2.1	7.1	0.1	0.3	0.6	1.1	58.0	0	10	4
U (μg L^{-1})	1.2	1.9	0.0	0.2	0.5	1.2	10.5	2.6	30	0
Mg (mg L^{-1})	14.1	9.1	0.0	6.0	12.8	21.5	34.6	1.3	50	0
Si (mg L^{-1})	7.0	3.1	0.1	5.6	7.2	9.2	13.5	9.3	-	NA
Ca (mg L^{-1})	27.7	19.5	1.2	20.0	23.2	38.4	86.2	16.0	200	0
Sr (mg L^{-1})	0.6	2.6	0.0	0.0	0.1	0.3	16.7	10.6	-	NA
Na (mg L^{-1})	25.7	49.2	0.0	11.8	15.6	20.2	313.0	2.6	200	2.6
K (mg L^{-1})	3.8	8.4	0.1	1.1	1.7	2.5	49.9	6.6	5	10.6

Table S2. Statistical summary of hydrochemistry data on dug wells sampled in 2014 (n = 29)

Variable	Mean	SD	Min	Q1	Median	Q3	Max	Cen (%)	Limit	% > Limit
Depth (m)	4.9	2.1	1.4	3.6	4.5	6.1	10.1	0	-	NA
Be (µg L^{-1})	-	-	-	-	-	-	0.2	89.6	-	NA
B (µg L^{-1})	23.3	13.4	10.5	13.7	17.6	29.5	63.7	0	1000	0
Al (µg L^{-1})	168.6	386.4	2.1	7.2	36.8	111.1	1731.3	0	200	13.8
Ti (µg L^{-1})	44.3	29.1	5.9	24.4	31.6	72.8	108.8	0	-	NA
V (µg L^{-1})	1.1	1.2	0.4	0.6	0.8	1.1	6.5	0	-	NA
Cr (µg L^{-1})	0.6	1.0	0.2	0.3	0.4	0.6	5.4	0	50	0
Mn (µg L^{-1})	34.1	133.6	0.2	0.7	3.4	11.4	718.9	0	50	6.9
Fe (µg L^{-1})	267.0	602.0	6.0	18.0	56.0	122.0	2239.0	0	200	17.2
Co (µg L^{-1}	0.2	0.2	0.0	0.1	0.1	0.2	0.9	17.2	-	NA
Ni (µg L^{-1}	2.1	1.0	0.5	1.4	2.2	2.7	4.9	0	20	0
Cu (µg L^{-1}	45.7	58.9	0.6	1.9	16.7	76.3	206.9	0	2000	0
Zn (µg L^{-1})	228.0	618.0	3.0	11.0	41.0	134.0	3264.0	0	5000	0
**As (µg L^{-1}**	**0.7**	**0.8**	**0.1**	**0.2**	**0.5**	**0.9**	**3.1**	**0**	10	**0**
Se (µg L^{-1})	0.6	0.4	0.2	0.3	0.5	0.7	2.6	0	10	0
Nb (µg L^{-1})	-	-	-	-	-	-	0.1	96.5	-	NA
Mo (µg L^{-1})	0.3	0.7	0.0	0.0	0.1	0.4	3.5	51.7	-	NA
Ag (µg L^{-1})	-	-	-	-	-	-	-	100	-	NA
Cd (µg L^{-1})	0.0	0.0	0.0	0.0	0.0	0.0	0.1	55.2	5	0
Sb (µg L^{-1})	0.2	0.4	0.0	0.0	0.0	0.1	1.7	72.4	5	0
Sn (µg L^{-1})	-	-	-	-	-	-	0.3	96.6	-	NA
Ba (µg L^{-1})	14.8	11.7	1.0	5.9	8.6	23.0	41.4	0	500	0
W (µg L^{-1})	-	-	-	-	-	-	0.3	96.6	-	NA
Pb (µg L^{-1})	2.0	4.5	0.1	0.3	0.8	2.0	24.2	0	10	3.4
U (µg L^{-1})	0.3	0.2	0.1	0.1	0.2	0.4	0.8	17.2	30	0
Mg (mg L^{-1})	10.1	4.7	2.3	6.6	10.6	13.1	18.9	0	50	0
Si (mg L^{-1})	5.5	4.2	0.1	0.6	5.7	8.2	14.9	20.6	-	NA
Ca (mg L^{-1})	26.9	19.7	4.1	10.5	19.9	39.7	77.5	31.3	200	0
Sr (mg L^{-1})	0.2	0.3	0.0	0.0	0.1	0.3	1.1	31.3	-	NA
Na (mg L^{-1})	13.0	11.0	0.0	0.1	12.6	19.7	40.8	13.8	200	0
K (mg L^{-1})	2.8	6.1	0.0	0.1	1.5	2.3	32.0	13.8	5	6.9

Table S3. Statistical summary of hydrochemistry data on springs sampled in 2014 (n = 3)

Variable	Mean	SD	Min	Q1	Median	Q3	Max	Cen (%)	Limit	% > Limit
Depth (m)	0.3	0.5	0.0	0.0	0.0	0.9	0.9	0	-	NA
Be (μg L^{-1})	-	-	-	-	-	-	0.2	33.3	-	NA
B (μg L^{-1})	12.0	2.1	10.3	10.3	11.5	14.3	14.3	0	1000	0
Al (μg L^{-1})	87.5	89.6	13.7	13.7	61.6	187.2	187.2	0	200	0
Ti (μg L^{-1})	15.0	11.9	3.9	3.9	13.5	27.6	27/64	0	-	NA
V (μg L^{-1})	0.8	0.5	0.3	0.3	0.7	1.3	1.3	0	-	NA
Cr (μg L^{-1})	0.4	0.1	0.2	0.2	0.3	0.5	0.5	0	50	0
Mn (μg L^{-1})	29.1	47.7	0.7	0.7	2.5	84.2	84.2	0	50	33.3
Fe (μg L^{-1})	132.7	159.1	19.6	19.6	63.7	314.6	314.6	0	200	33.3
Co (μg L^{-1}	-	-	-	-	-	-	0.1	33.3	-	NA
Ni (μg L^{-1}	0.9	0.5	0.5	0.5	0.6	1.5	1.5	0	20	0
Cu (μg L^{-1}	0.9	0.3	0.6	0.6	1.0	1.2	1.2	0	2000	0
Zn (μg L^{-1})	3.1	0.9	2.2	2.2	3.0	4.0	4.0	0	5000	0
**As (μg L^{-1}**	**0.3**	**0.1**	**0.2**	**0.2**	**0.3**	**0.3**	**0.3**	**0**	10	**0**
Se (μg L^{-1})	0.3	0.1	0.2	0.2	0.3	0.5	0.5	0	10	0
Nb (μg L^{-1})	-	-	-	-	-	-	-	100.0	-	NA
Mo (μg L^{-1})	-	-	-	-	-	-	0.4	66.6	-	NA
Ag (μg L^{-1})	-	-	-	-	-	-	-	100.0	-	NA
Cd (μg L^{-1})	-	-	-	-	-	-	-	100.0	5	0
Sb (μg L^{-1})	-	-	-	-	-	-	-	100.0	5	0
Sn (μg L^{-1})	-	-	-	-	-	-	-	100.0	-	NA
Ba (μg L^{-1})	3.9	3.9	1.6	1.6	1.7	8.3	8.3	0	500	0
W (μg L^{-1})	-	-	-	-	-	-	-	100.0	-	NA
Pb (μg L^{-1})	-	-	-	-		-	0.2	33.3	10	0
U (μg L^{-1})	0.1	0.1	0.1	0.1	0.1	0.2	0.2	0	30	0
Mg (mg L^{-1})	5.5	3.8	2.5	2.5	4.2	9.8	9.8	0	50	0
Si (mg L^{-1})	-	-	-	-	-	-	5.6	33.3	-	NA
Ca (mg L^{-1})	-	-	-	-	-	-	42.5	33.3	200	0
Sr (mg L^{-1})	-	-	-	-	-	-	0.2	33.3	-	NA
Na (mg L^{-1})	-	-	-	-	-	-	31.8	33.3	200	0
K (mg L^{-1})	-	-	-	-	-	-	9.1	33.3	5	33.3

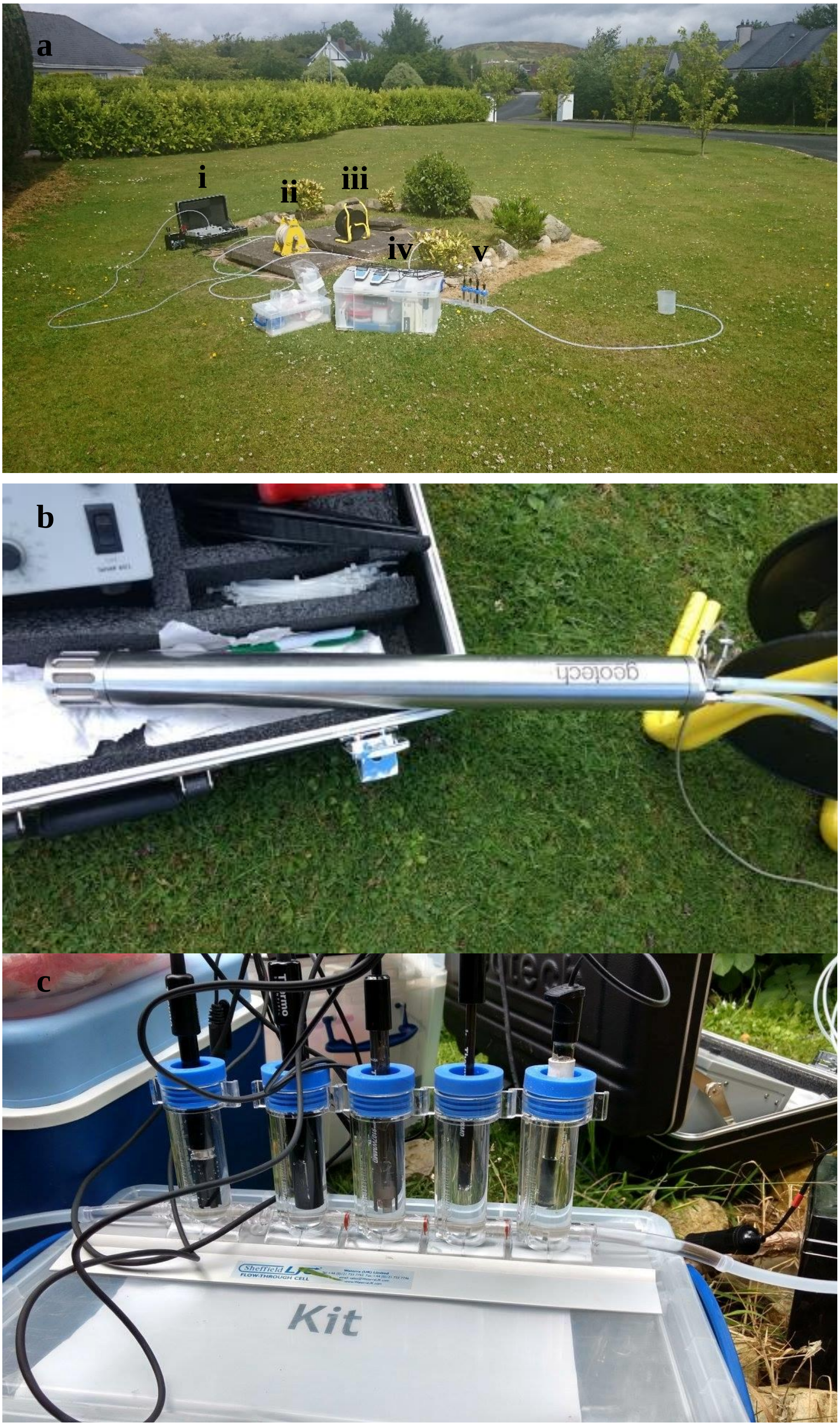
a
i
ii
iii
iv
v
b
geotech
c
FLOW-THROUGH CELL
Kit

Fig. S2. Groundwater sampling set up for trace element sampling in (**a**) Louth (BH-24) with the following equipment: i) controller unit, ii) dip meter, iii) safety cable iv) electrochemistry probes, and v) flow-through cell, (**b**) detailed image showing the bladder pump unit with air and sample tubing with a safely line (Dyneema®) attached, and (**c**) LFC groundwater flow-cell with groundwater moving from the right inlet to the left outlet. Electrochemical probes from right to left: Eh, temperature, pH (with temperature), conductivity and dissolved oxygen. (source: authors own image).

Note 2 – Sample bottle decontamination

In this work, the following washing procedure was used sample bottle decontamination which was modified from a GEOTRACES protocol (Cutter et al. 2010). In this study, HCl was used as the acid first as traces of chloride may interfere with spectroscopic determination of arsenic.

1) LDPE bottles (60, 125, 250, 500 and 1000 cm^3, Fisher Scientific, Dublin) were rinsed out three times with ultrapure water (18 MΩ, Millipore Elix and Milli-Q, Ireland) and left in 1% detergent solution (Citronrox, Lennox, Ireland) for 24 hours.
2) Bottles were rinsed out of detergent several times to remove traces of detergent. Bottles were then left soaked in 40% (v/v) HCl (TraceMetal™ grade, Fisher Chemical, Fisher Scientific, Ireland) for a week.
3) Bottles were then emptied of HCl and rinsed to remove traces of acid. Following this, 40% (v/v) of HNO_3 (TraceMetal™ grade, Fisher Chemical, Fisher Scientific, Ireland) was soaked in the bottles for a week and then removed and rinsed.
4) Finally, bottles were soaked in 2% ultrapure HNO_3 (v/v) (Optima™, Fisher Chemical, Fisher Scientific, Ireland) for 48 hours and rinsed.
5) Bottles were then filled with Milli-Q water (18 MΩ) and stored in bags (Bryson Packaging™ Minigrip™ PE Bags, Fisher Scientific, Ireland) until sampling occurred.
6) Washings of water along the cleaning procedure were collected and analysed for trace element concentrations.

Table S4. Operating conditions for ICP-MS and HPLC for total metal and speciation analysis

	Settings	
Analytical system	Total/dissolved trace elements	Speciation analysis
HPLC (Perkin-Elmer series 200)		
Guard column		
Analytical column		Hamilton PRP-X100 (4.1 x 250 mm, 5 µm)
Mobile phase		4 mM NH_4NO_3 and 60 mM NH_4NO_3 (pH 8.67)
Flow rate (1 cm^3 min^{-1})		1
Injection volume (µL)		100
Acquisition time (min)		15
ICP-MS (PE Elan DRC-e)		
RF Power (W)	1200	1200
Lens voltage (V)	8.25	8.25
Plasma gas flow (L min^{-1})	15.00	15.00
Auxiliary gas flow (L min^{-1})	1.00	1.00
Nebuliser gas flow (L min^{-1})	0.93-0.97	0.93-0.97
Nebuliser type	Cyclonic spray chamber	Cyclonic spray chamber
Sampling cone	Nickel	Nickel
Skimmer cone	Nickel	Nickel
Dwell time (ms)	50	50
Scan mode	Peak hopping	Peak hopping
Internal standard (100 µg L^{-1})	^{115}In	^{115}In
DRC reaction gas	CH_4/O_2	O_2
Units	Counts per second	Counts per second
Carrier gas	Ar (99.999%)	Ar (99.999%)
Reaction gas	O_2 (99.9995%), CH_4 (99.99%)	O_2 (99.9995%),

Table S5. ICP-MS analytical figures of merit

Analyte	Units	LOD	LOQ	ICP-MS mode	Calibration range
^{9}Be	µg L^{-1}	0.05	0.12	Standard	0.1-200
^{11}B	µg L^{-1}	0.35	0.68	Standard	0.1-200
^{23}Na	mg L^{-1}	0.01	0.02	Standard (Majors)	0.1-200
^{24}Mg	mg L^{-1}	0.01	0.02	Standard (Majors)	0.1-200
^{27}Al	µg L^{-1}	0.05	0.12	Standard	0.1-200
^{28}Si	mg L^{-1}	0.01	0.06	Standard (Majors)	0.025-50
^{39}K	mg L^{-1}	0.01	0.02	Standard (Majors)	0.025-50
^{43}Ca	mg L^{-1}	0.01	0.09	Standard (Majors)	0.1-200
^{47}Ti	µg L^{-1}	0.01	0.16	Standard	0.01-20
^{51}V	µg L^{-1}	0.06	0.12	Standard	0.1-200
^{52}Cr	µg L^{-1}	0.02	0.06	DRC (CH_4)	0.1-200
^{55}Mn	µg L^{-1}	0.05	0.10	Standard	0.1-200
^{56}Fe	µg L^{-1}	0.02	0.33	DRC (CH_4)	0.1-200
^{59}Co	µg L^{-1}	0.03	0.09	Standard	0.1-200
^{60}Ni	µg L^{-1}	0.19	0.41	Standard	0.1-200
^{63}Cu	µg L^{-1}	0.08	0.17	Standard	0.1-200
^{66}Zn	µg L^{-1}	0.04	0.17	DRC (CH_4)	0.1-200
^{75}As	µg L^{-1}	0.03	0.07	DRC (O_2)	0.1-200
^{82}Se	µg L^{-1}	0.01	0.09	DRC (CH_4)	0.1-200
^{88}Sr	mg L^{-1}	0.002	0.004	Standard (Majors)	0.025-50
^{93}Nb	µg L^{-1}	0.03	0.07	Standard	0.01-20
^{98}Mo	µg L^{-1}	0.06	0.14	Standard	0.01-20
^{107}Ag	µg L^{-1}	0.05	0.09	Standard	0.1-200
^{111}Cd	µg L^{-1}	0.01	0.03	Standard	0.1-200
^{118}Sn	µg L^{-1}	0.03	0.10	Standard	0.01-20
^{121}Sb	µg L^{-1}	0.05	0.10	Standard	0.01-20
^{130}Te	µg L^{-1}	0.004	0.05	Standard	0.01-20
^{138}Ba	µg L^{-1}	0.05	0.10	Standard	0.1-200
^{184}W	µg L^{-1}	0.03	0.07	Standard	0.01-20
^{205}Tl	µg L^{-1}	0.03	0.06	Standard	0.1-200
^{208}Pb	µg L^{-1}	0.03	0.07	Standard	0.1-200
^{209}Bi	µg L^{-1}	0.07	0.10	Standard	0.1-200
^{238}U	µg L^{-1}	0.03	0.07	Standard	0.1-200

Table S6. HPLC programme using 4 mM NH_4NO_3 (A) and 60 mM NH_4NO_3 (B) (between each step between 0.25 and 1 minute was allowed to change from 100% of one solvent to another. The last step of the programme was extended to allow for adequate column equilibration prior to subsequent injection).

Mobile phase	Time (minutes)				
	0-2	3-6.5	7.5-10.75	11-13	13.25-17
A (%)	100	0	100	0	100
B (%)	0	100	0	100	0

Table S7. Recovery of CRM 1643f from 2016 (n=6) showing average concentration and average recovery. Values are represented by mean ± standard deviation. All parameters reported in µg L^{-1} except for Mg, Na, Sr, K and Ca where parameters are reported in mg L^{-1}.

Parameter	Certified value	Average concentration	Average Recovery (%)
B	152.3	175.2±33.0	115.0±21.7
Al	133.8	165.7±35.0	123.8±26.1
V	36.07	37.9±1.9	105.0±5.3
Cr	18.5	20.2±1.6	108.9±8.4
Mn	37.14	37.8±1.0	101.8±2.7
Fe	93.44	100.1±7.5	107.1±8.0
Co	25.3	25.5±0.9	100.9±3.7
Ni	59.8	60.6±2.7	101.3±4.6
Cu	21.66	21.5±1.1	99.2±5.0
Zn	74.4	79.0±6.0	106.2±8.1
As	57.42	61.6±0.8	107.4±1.4
Se	11.7	14.0±1.5	119.9±12.7
Mo	115.3	117.3±2.8	101.7±2.4
Ag	0.9703	0.5±0.0	46.9±4.4
Cd	5.89	6.5±0.2	110.2±3.7
Sb	55.45	58.7±1.7	105.9±3.1
Te	0.977	0.8±0.2	79.3±18.4
Ba	518.2	512.8±25.4	99.0±4.9
Tl	6.892	7.1±0.4	103.3±6.1
Pb	18.488	19.2±1.2	103.8±6.3
Bi	12.62	11.8±1.0	93.8±7.9
Mg	7.454	7.9±0.6	105.4±8.1
Na	18.83	21.0±1.8	111.6±9.6
Sr	0.314	0.3±0.0	106.1±6.0
K	1.9326	2.0±0.2	106.0±9.7
Ca	29.43	27.0±2.2	91.8±7.5

Table S8. Statistical summary of hydrochemistry data on bedrock boreholes sampled in 2016 (n=17)

Variable	Mean	SD	Min	Q1	Median	Q3	Max	Cen (%)	Limit	% > Limit
Depth (m)	86.9	30.9	17.3	68.5	91.4	110.3	148.8	0	-	NA
SWL (m)	6.5	6.6	1.8	3.3	5.0	5.3	30.2	0	-	NA
Temp (°C)	13.7	2.1	10.9	12.1	13.5	14.7	18.6	0	-	NA
C (µS cm^{-1})	338.3	143.3	88.6	233.2	322.8	415.3	650.0	0	2500	0
TDS (mg L^{-1})	233.5	98.9	61.2.	160.9	222.8	286.6	448.5	0	-	NA
pH	7.7	0.3	6.9	7.4	7.8	8.0	8.3	0	≥6.5 and ≤9.5	0
Eh (mV)	300.6	139.1	-121.5	248.6	300.7	420.5	460.3	0	-	NA
DO (mg L^{-1})	4.2	6.9	0.1	0.7	1.4	5.8	28.5	0	-	NA
Cl^- (mg L^{-1})	17.4	14.2	0.3	11.1	12.1	18.5	62.3	0	250	0
SO_4^{2-} (mg L^{-1})	15.6	6.4	8.0	10.0	13.0	21.5	28.0	0	250	0
F^- (mg L^{-1})	0.2	0.1	0.1	0.1	0.2	0.2	0.3	0	1.5	0
Alk (mg L^{-1})	118.7	45.4	37.7	80.6	119.8	157.9	226.5	0	-	NA
B (µg L^{-1})	10.3	7.8	5.1	6.0	6.8	12.0	30.3	0	1000	0
Al (µg L^{-1})	1.8	1.8	0.4	0.8	1.1	2.5	8.1	0	200	0
Ti (µg L^{-1})	0.5	0.4	0.1	0.3	0.3	0.9	1.4	5.88	-	NA
V (µg L^{-1})	1.3	1.7	0.0	0.2	0.5	1.8	5.3	11.7	-	NA
Cr (µg L^{-1})	0.2	0.2	0.0	0.1	0.2	0.3	0.7	5.88	50	0
Mn (µg L^{-1})	4.3	13.8	0.0	0.1	0.4	0.9	57.3	11.7	50	5.88
Fe (µg L^{-1})	4.5	10.4	0.0	0.0	0.4	1.3	32.8	41.1	200	0
Co (µg L^{-1}	0.1	0.0	0.0	0.1	0.1	0.1	0.1	64.7	-	NA
Ni (µg L^{-1}	1.5	0.7	0.6	1.0	1.2	2.1	3.5	0	20	0
Cu (µg L^{-1}	0.9	0.7	0.2	0.4	0.7	1.1	2.9	0	2000	0
Zn (µg L^{-1})	2.5	2.1	0.4	0.9	1.7	3.7	7.5	0	5000	0
**As (µg L^{-1})**	**20.7**	**19.9**	**0.1**	**4.9**	**15.5**	**28.8**	**74.0**	**0**	**10**	**70.5**
As^{III} (µg L^{-1})	0.2	0.2	0.0	0.0	0.1	0.3	0.6	0	10	0
As^{V} (µg L^{-1})	16.9	16.9	0.1	3.9	11.8	25.3	64.3	0	10	64.7
DMA (µg L^{-1})	0.7	0.5	0.1	0.3	0.5	0.9	2.3	0	10	0
MA (µg L^{-1})	0.0	0.0	0.0	0.0	0.0	0.0	0.1	0	10	0
Se (µg L^{-1})	0.4	1.0	0.0	0.0	0.1	0.3	4.3	58.8	10	0
Mo (µg L^{-1})	2.4	2.3	0.1	0.6	1.9	3.5	8.0	5.8	-	NA
Ag (µg L^{-1})	-	-	-	-	-	-	-	100	-	NA
Cd (µg L^{-1})	-	-	-	-	-	-	0.0	94.2	5	0
Sn (µg L^{-1})	0.1	0.1	0.0	0.1	0.1	0.2	0.3	64.7	-	NA
Sb (µg L^{-1})	0.6	0.8	0.0	0.1	0.3	0.6	3.3	11.7	5	0
Ba (µg L^{-1})	64.1	81.9	0.2	0.7	13.7	114.6	284.3	0	500	0
W (µg L^{-1})	-	-	-	-	-	-	0.2	82.3	-	NA
Pb (µg L^{-1})	-	-	-	-	-	-	1.4	82.3	10	0
U (µg L^{-1})	1.6	2.3	0.0	0.2	0.6	2.1	7.6	17.6	30	0
Mg (mg L^{-1})	13.7	10.1	0.2	3.4	15.0	20.8	32.1	0	50	0
Si (mg L^{-1})	6.2	1.9	0.4	5.9	6.3	6.7	10.6	0	-	NA
Ca (mg L^{-1})	30.8	15.7	10.2	21.8	24.9	35.9	81.8	0	200	0
Na (mg L^{-1})	14.2	4.6	3.9	11.0	14.6	17.1	22.7	0	200	0
Sr (mg L^{-1})	0.4	0.4	0.0	0.0	0.5	0.6	1.1	0	-	NA
K (mg L^{-1})	1.5	0.7	0.2	1.1	1.4	1.7	3.0	0	5	0

Table S9. Statistical summary of hydrochemistry data on dug wells sampled in 2016 (n=3)

Variable	Mean	SD	Min	Q1	Median	Q3	Max	Cen (%)	Limit	% > Limit
Depth (m)	3.7	0.3	3.5	3.5	3.6	4.1	4.1	0	-	NA
SWL (m)	2.7	0.9	1.7	1.7	3.2	3.3	3.3	0	-	NA
Temp (°C)	14.7	2.9	12.2	12.2	13.9	18.0	18.0	0	-	NA
C (µS cm^{-1})	623.0	521.0	224.0	224.0	433.0	1213.0	1213.0	0	2500	0
TDS (mg L^{-1})	430.0	360.0	154.0	154.0	299.0	837.0	837.0	0	-	0
pH	6.8	0.3	6.5	6.5	6.8	7.1	7.1	0	≥6.5 and ≤9.5	33.3
Eh (mV)	247.0	271.0	-52.0	-52.0	316.0	476.0	476.0	0	-	NA
DO (mg L^{-1})	5.2	3.1	2.2	2.2	5.3	8.3	8.3	0	-	NA
Cl^- (mg L^{-1})	118.2	168.3	8.6	8.6	33.9	312.0	312.0	0	250	33.3
SO_4^{2-} (mg L^{-1})	14.7	6.0	9.0	9.0	14.0	21.0	21.0	0	250	0
F^- (mg L^{-1})	0.1	0.0	0.1	0.1	0.1	0.2	0.2	0	1.5	0
Alk (mg L^{-1})	99.6	29.3	73.0	73.0	94.7	131.0	131.0	0	-	NA
B (µg L^{-1})	20.7	14.7	7.1	7.1	18.7	36.3	26.3	0	1000	0
Al (µg L^{-1})	3.1	2.4	1.5	1.5	2.0	5.9	5.9	0	200	0
Ti (µg L^{-1})	0.6	0.5	0.3	0.3	0.4	1.2	1.2	0	-	NA
V (µg L^{-1})	1.4	0.6	0.8	0.8	1.7	1.8	1.8	0	-	NA
Cr (µg L^{-1})	0.3	0.1	0.2	0.2	0.2	0.5	0.4	0	50	0
Mn (µg L^{-1})	48.0	59.0	6.7	6.7	21.7	115.5	115.5	0	50	33.3
Fe (µg L^{-1})	47.0	57.3	5.5	5.5	23.2	112.3	112.3	0	200	0
Co (µg L^{-1}	-	-	-	-	-	-	0.5	33.3	-	NA
Ni (µg L^{-1}	2.2	1.0	1.2	1.2	2.2	3.3	3.3	0	20	0
Cu (µg L^{-1}	2.9	2.3	0.9	0.9	2.4	5.3	5.3	0	2000	0
Zn (µg L^{-1})	6.7	4.2	2.3	2.3	7.0	10.7	10.7	0	5000	0
**As (µg L^{-1})**	**0.9**	**1.0**	**0.3**	**0.3**	**0.4**	**2.0**	**2.0**	**0**	10	**0**
As^{III} (µg L^{-1})	0.2	0.3	0.0	0.0	0.1	0.5	0.5	0	10	0
As^{V} (µg L^{-1})	0.7	0.9	0.1	0.1	0.3	1.7	1.7	0	10	0
DMA (µg L^{-1})	0.2	0.0	0.2	0.2	0.2	0.2	0.2	0	10	0
MA (µg L^{-1})	0.0	0.0	0.0	0.0	0.0	0.1	0.1	0	10	0
Se (µg L^{-1})	-	-	-	-	-	-	-	100	10	0
Mo (µg L^{-1})	-	-	-	-	-	-	1.0	33.3	-	NA
Ag (µg L^{-1})	-	-	-	-	-	-	0.3	66.6	-	NA
Cd (µg L^{-1})	-	-	-	-	-	-	0.1	66.6	5	0
Sn (µg L^{-1})	-	-	-	-	-	-	0.3	66.6	-	NA
Sb (µg L^{-1})	-	-	-	-	-	-	0.2	66.6	5	0
Ba (µg L^{-1})	14.9	15.9	5.3	5.3	6.1	33.3	33.3	0	500	0
W (µg L^{-1})	-	-	-	-	-	-	-	100.0	-	NA
Pb (µg L^{-1})	-	-	-	-	-	-	-	100.0	10	0
U (µg L^{-1})	0.3	0.2	0.1	0.1	0.3	0.5	0.5	0	30	0
Mg (mg L^{-1})	9.8	7.9	3.0	3.0	8.0	18.6	18.6	0	50	0
Si (mg L^{-1})	6.3	1.3	5.4	5.4	5.6	7.7	7.7	0	-	NA
Ca (mg L^{-1})	46.8	21.6	25.6	25.6	46.1	68.7	68.7	0	200	0
Na (mg L^{-1})	52.1	54.1	12.5	12.5	30.2	113.7	113.7	0	200	0
Sr (mg L^{-1})	0.2	0.2	0.0	0.0	0.1	0.4	0,364	0	-	NA
K (mg L^{-1})	3.5	3.7	1.1	1.1	1.7	7.8	7.8	0	5	33.3

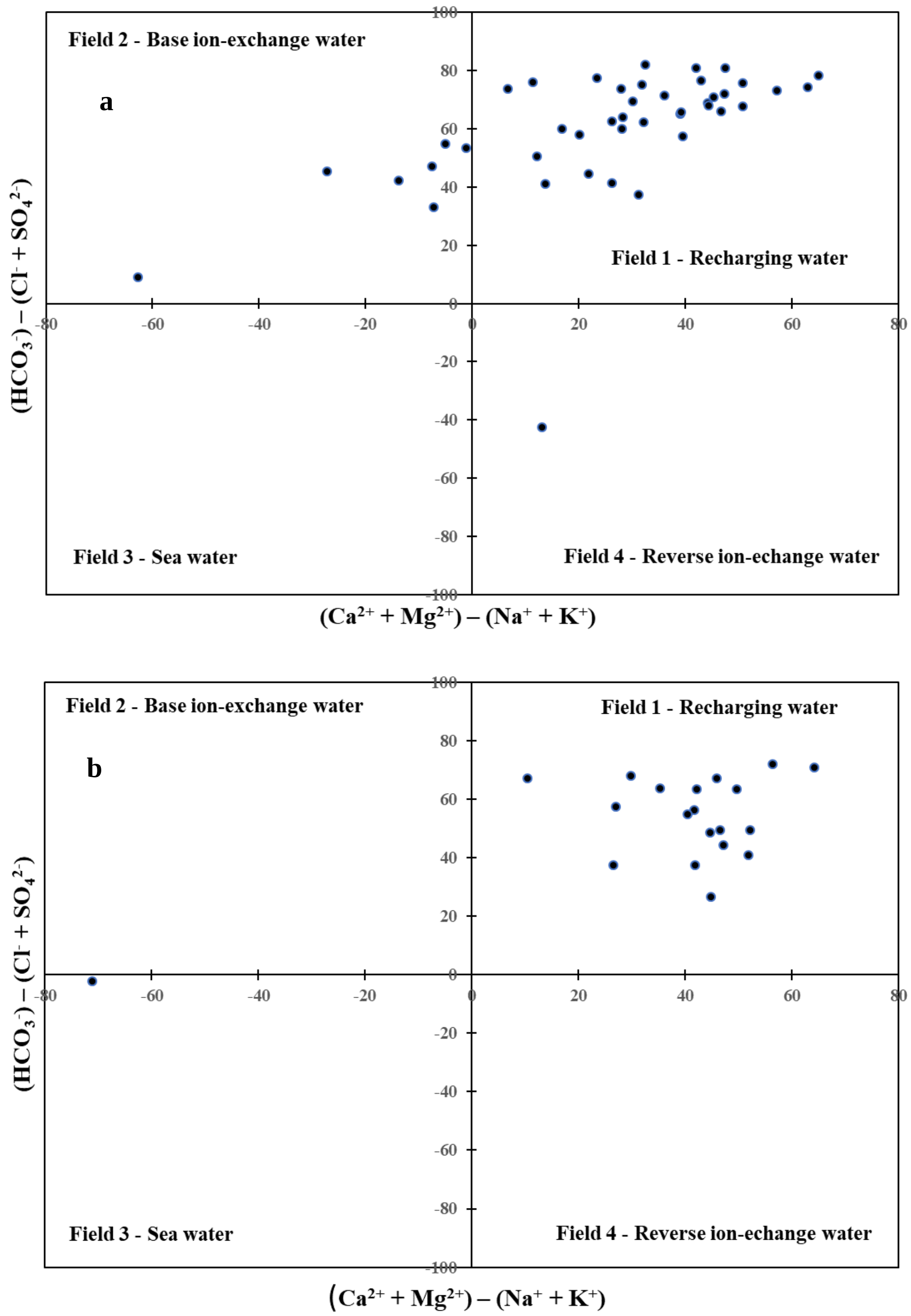

Fig. S3. Chadha's diagram for (**a**) 2015 and (**b**) 2016 data.

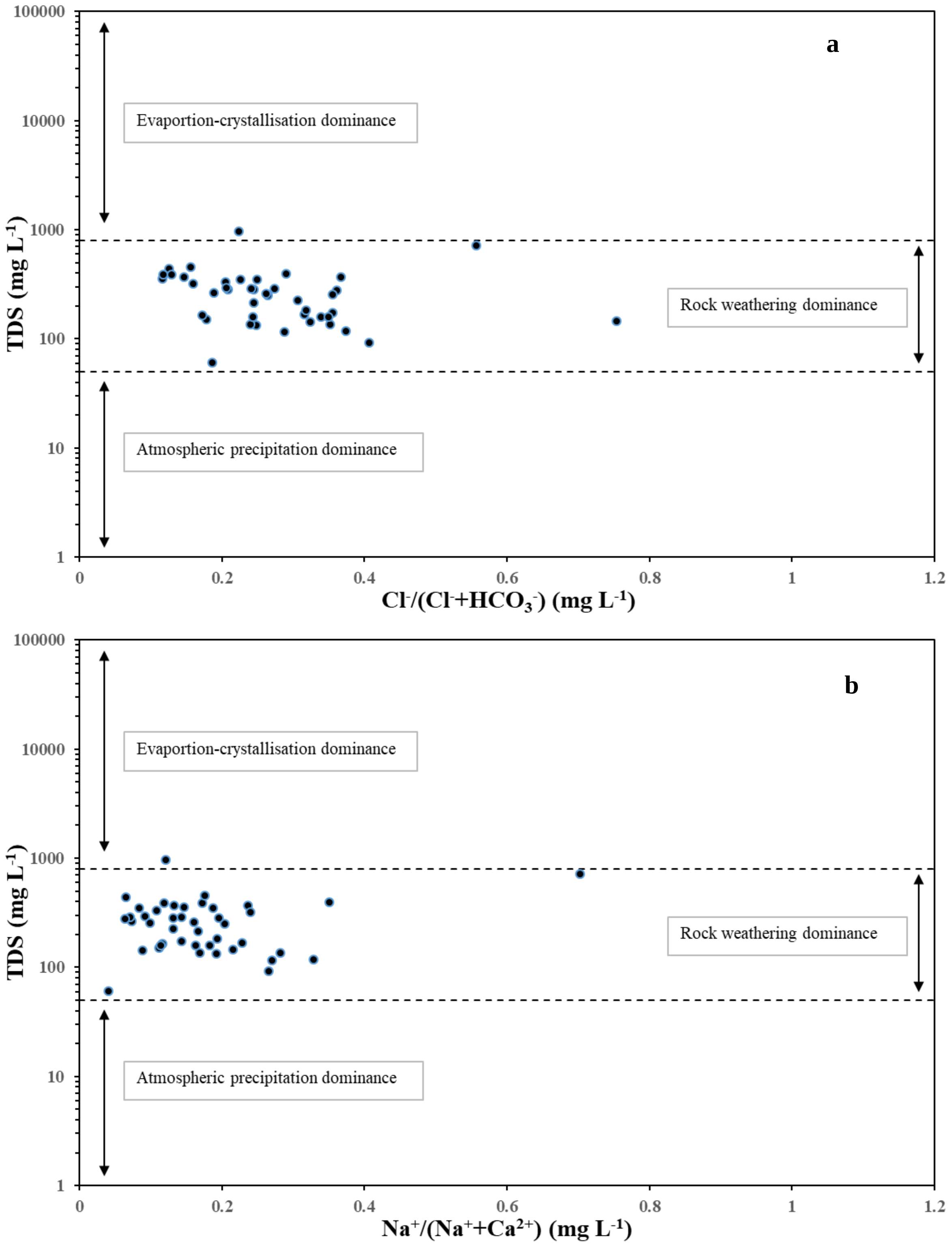

Fig. S4. Gibbs diagram illustrating the mechanisms controlling groundwater geochemistry for (**a**) 2015 anions, (**b**) 2015 cations, (**c**) 2016 anions, and (**d**) 2016 cations.

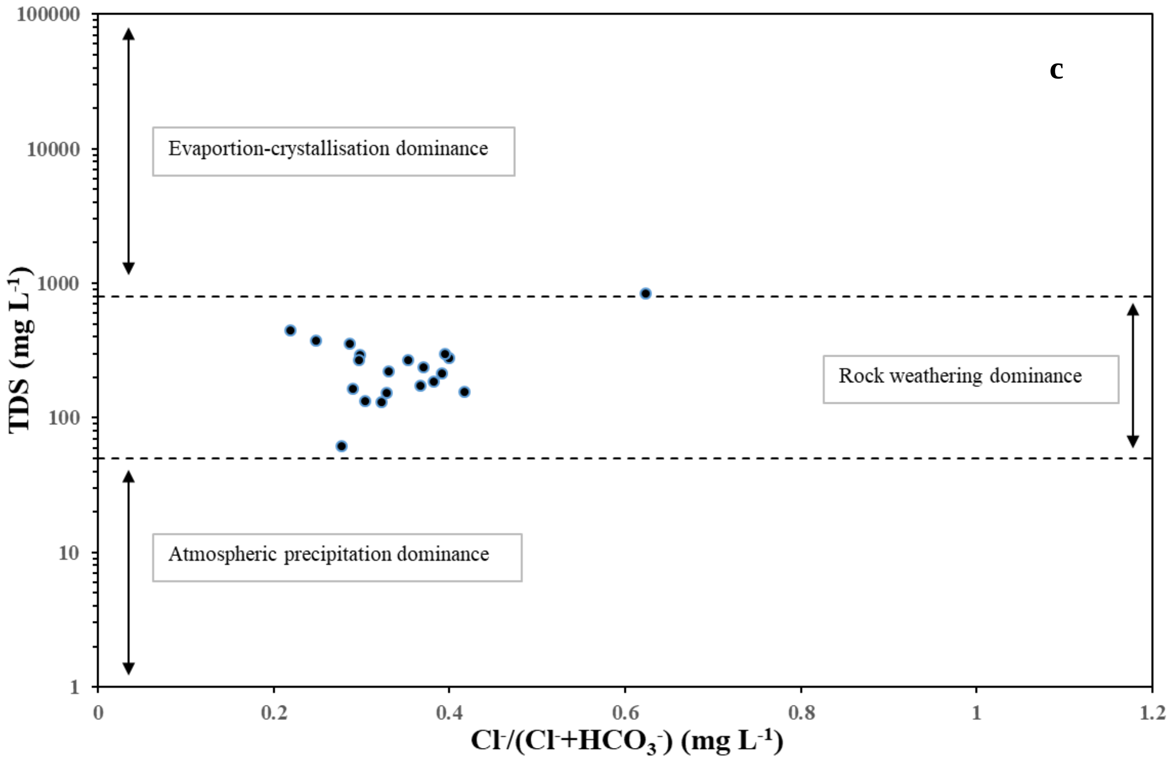

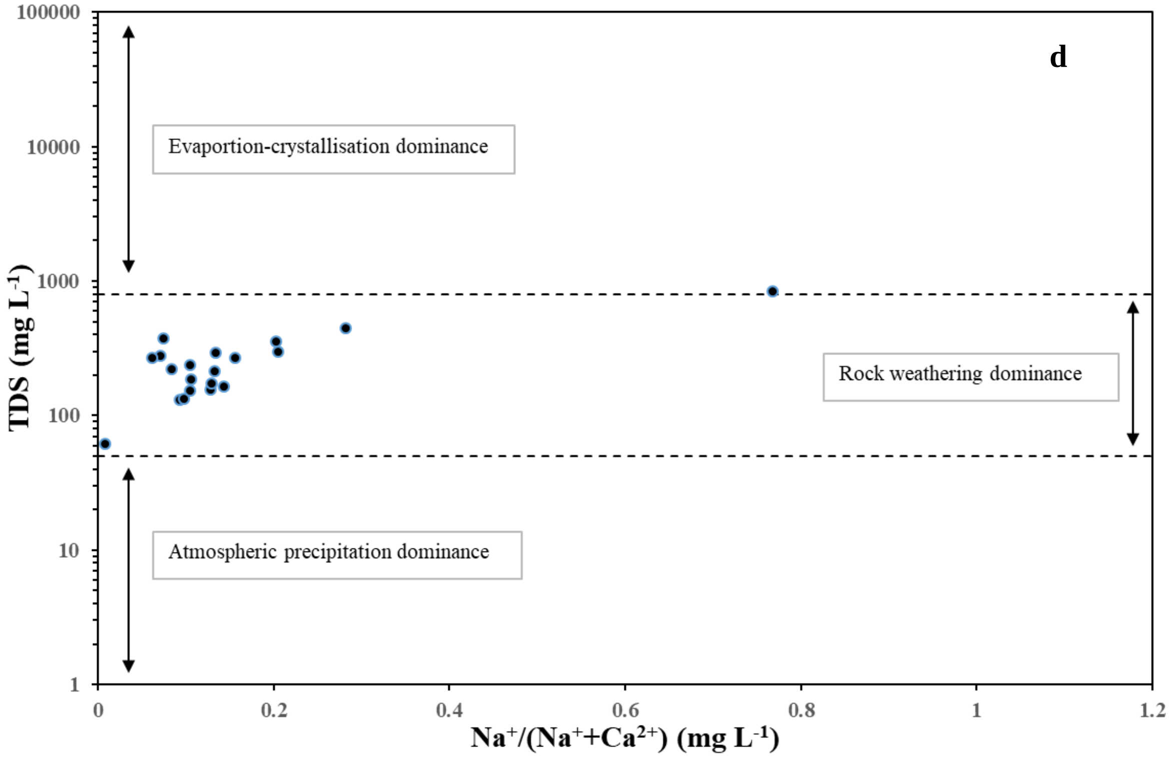

Fig. S4. *(continued).*

Table S10. Comparison of filtered and unfiltered trace elements in groundwater (n = 43 for 2015 and n = 20 for 2016).

	2015				2016			
	Tau	p-value	S	Diff (%)	Tau	p-value	S	Diff (%)
B	0.94	≈ 0.000	0.902	9.8	0.80	≈ 0.000	0.925	7.5
Al	0.50	≈ 0.000	0.729	27.1	0.37	0.023	0.108	89.2
Ti	0.29	0.005	0.618	38.2	0.43	0.007	0.525	47.5
V	0.85	≈ 0.000	0.927	7.3	0.96	≈ 0.000	0.974	2.6
Cr	0.47	≈ 0.000	0.870	13.0	0.84	≈ 0.000	0.919	8.1
Mn	0.71	0.000	0.415	58.5	0.70	≈ 0.000	0.688	31.2
Fe	0.51	≈ 0.000	0.124	87.6	0.44	0.008	0.049	95.1
Co	0.36	≈ 0.000	0.998	0.2	0.86	≈ 0.000	0.996	0.4
Ni	0.85	0.000	0.933	6.7	0.77	≈ 0.000	0.710	29.0
Cu	0.81	0.000	0.950	5.0	0.86	≈ 0.000	0.897	10.3
Zn	0.85	0.000	0.898	10.2	0.84	≈ 0.000	0.953	4.7
**As**	**0.93**	**0.000**	**0.876**	**12.4**	**0.96**	**0.000**	**0.978**	**2.2**
Se	0.77	0.000	0.972	2.8	0.83	≈ 0.000	0.962	3.8
Mo	0.98	0.000	0.987	1.3	0.96	≈ 0.000	0.986	1.4
Sn	-	-	-	-	0.55	0.001	0.797	20.3
Sb	0.98	0.000	0.904	9.6	0.95	≈ 0.000	0.963	3.7
Ba	0.96	0.000	0.982	1.8	1.0	0.000	0.970	3.0
W	0.98	0.000	0.919	8.1	-	-	-	-
Pb	0.34	0.004	0.626	37.4	0.33	0.054	0.838	16.2
U	0.97	0.000	0.986	1.4	0.86	0.000	0.914	8.6
Mg	0.96	0.000	0.880	12.0	0.98	0.000	0.952	4.8
Si	0.86	0.000	0.823	17.7	0.77	≈ 0.000	0.884	11.6
Ca	0.86	0.000	0.750	25.0	0.95	0.000	0.934	6.6
Na	0.92	0.000	0.921	7.9	0.90	0.000	0.866	13.4
Sr	0.96	0.000	0.872	12.8	0.90	0.000	0.946	5.4
K	0.86	0.000	0.848	15.2	0.93	0.000	0.873	12.7

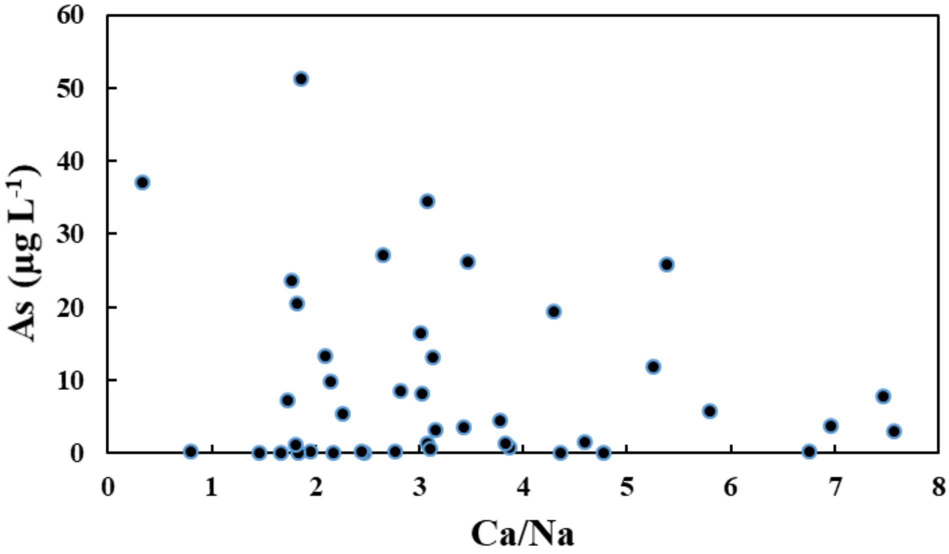

Fig. S5. Relationship between arsenic concentrations and Ca/Na ratios in groundwaters for 2015 data.

Table S11. SIs for several Fe, Mn, Al and Cu minerals (2015 and 2016)

2015<br>Name	Formula	Median	Average	SD	Min	Max
$Ba_3(AsO_4)_2$	$Ba_3(AsO_4)_2$	1.88	3.33	4.81	-4.65	11.41
Basaluminite	$Al_4(OH)10SO_4$	-4.13	-3.75	3.89	-10.28	6.44
Boehmite	AlOOH	-0.36	-0.24	0.63	-1.41	1.79
Calcite	$CaCO_3$	-0.25	-0.59	0.78	-2.47	0.49
Cupric ferrite	$CuFe_2O_4$	8.81	9.27	1.87	4.68	14.60
Cuprous ferrite	$CuFeO_2$	6.19	6.71	1.80	4.19	13.07
Diaspore	AlOOH	1.48	1.59	0.63	0.42	3.61
Dolomite	$CaMg(CO_3)_2$	-1.07	-1.70	1.69	-5.53	0.55
$Fe(OH)_2$.7Cl.3	$Fe(OH)_2$.7Cl0.3	5.35	5.46	0.91	4.03	7.85
$Fe(OH)_3$(a)	$Fe(OH)_3$	0.52	0.72	0.86	-1.04	3.01
Fe_3(OH) 8	Fe_3(OH) 8	-6.65	-5.64	3.37	-9.47	4.89
Gibbsite	$Al(OH)_3$	0.33	0.43	0.63	-0.74	2.45
Goethite	FeOOH	5.95	6.10	0.85	4.28	8.39
Gypsum	$CaSO_4:2H_2O$	-2.65	-2.61	0.28	-3.11	-1.95
Halite	NaCl	-8.12	-8.11	0.48	-9.87	-6.18
Hematite	Fe_2O_3	13.84	14.14	1.70	10.50	18.72
Kaolinite	$Al_2Si_2O_5(OH)_4$	1.68	1.96	1.29	-0.37	6.59
Kmica	$KAl_3Si_3O_{10}(OH)_2$	3.89	4.08	1.73	1.15	10.74
Maghemite	Fe_2O_3	4.44	4.85	1.72	1.31	9.41
Magnetite	Fe_3O_4	7.93	9.05	3.34	5.06	19.63
Malachite	$Cu_2(OH)_2CO_3$	-5.49	-5.37	0.84	-7.04	-1.86
Pyrophyllite	$Al_2Si_4O_{10}(OH)_2$	2.51	2.61	1.57	0.28	9.84
Scorodite	$FeAsO_4:2H_2O$	-8.61	-8.57	0.99	-10.12	-5.57
Siderite	$FeCO_3$	-6.59	-6.40	2.15	-10.13	1.17
Talc	$Mg_3Si_4O_{10}(OH)_2$	-5.26	-5.84	4.33	-13.90	-0.02
2016<br>Name	Formula	Median	Average	SD	Min	Max
Adularia	$KAlSi_3O_8$	4.93	4.82	0.77	1.94	5.74
Albite	$NaAlSi_3O_8$	3.33	3.27	0.91	-0.30	4.38
$Ba_3(AsO_4)_2$	$Ba_3(AsO_4)_2$	12.94	13.05	4.58	2.85	19.01
Barite	$BaSO_4$	1.84	1.73	1.09	-0.13	3.25
Basaluminite	$Al_4(OH)10SO_4$	-4.59	-4.21	2.89	-8.18	0.89
Boehmite	AlOOH	0.34	0.46	0.57	-0.32	1.55
Calcite	$CaCO_3$	2.58	2.35	0.49	1.46	2.98
Cupric ferrite	$CuFe_2O_4$	12.12	11.45	4.67	-2.43	16.51
Cuprous ferrite	$CuFeO_2$	10.28	10.40	1.65	7.28	14.16
Diaspore	AlOOH	2.04	2.16	0.57	1.38	3.26
Dolomite	$CaMg(CO_3)_2$	5.04	4.28	1.29	1.42	5.62
$Fe(OH)_2$.7Cl.3	$Fe(OH)_2$.7Cl0.3	6.17	6.04	1.84	1.13	8.80
$Fe(OH)_3$(a)	$Fe(OH)_3$	1.11	0.97	1.85	-4.28	3.05
Fe_3(OH) 8	Fe_3(OH) 8	-1.72	-2.33	3.54	-11.25	4.28
Gibbsite	$Al(OH)_3$	0.81	0.93	0.57	0.15	2.03
Goethite	FeOOH	7.00	6.86	1.85	1.61	8.94
Gypsum	$CaSO_4:2H_2O$	-0.18	-0.17	0.18	-0.53	0.10
Halite	NaCl	-5.51	-5.49	0.77	-7.67	-3.37
Hematite	Fe_2O_3	16.02	15.74	3.69	5.24	19.89
Jarosite-Na	$NaFe_3(SO_4)_2(OH)_6$	-6.64	-6.85	5.76	-22.08	3.38
Kaolinite	$Al_2Si_2O_5(OH)_4$	6.00	6.17	1.10	4.70	8.36
Kmica	$KAl_3Si_3O_{10}(OH)_2$	12.06	12.29	1.41	10.37	15.23
Maghemite	Fe_2O_3	5.63	5.34	3.69	-5.16	9.50
Magnetite	Fe_3O_4	14.83	13.05	5.19	1.84	20.77
Malachite	$Cu_2(OH)_2CO_3$	-2.13	-2.67	2.01	-8.56	-0.58
Pyrophyllite	$Al_2Si_4O_{10}(OH)_2$	12.20	12.10	1.33	8.65	14.30
Scorodite	$FeAsO_4:2H_2O$	-6.04	-6.56	2.47	-14.19	-3.92
Siderite	$FeCO_3$	-1.48	-1.99	1.77	-5.55	0.53
Talc	$Mg_3Si_4O_{10}(OH)_2$	8.51	6.77	3.96	-3.35	10.80

Table S12. PCA scores for ranked data in 2016. Scores highlighted in bold are significant.

Variable	PC1	PC2	PC3	PC4	PC5	PC6	PC7	PC8	PC9	PC10
Eigenvalue	10.8	8.7	5.0	3.5	3.3	2.5	1.6	1.4	1.3	1.0
Proportion (%)	25.1	20.2	11.5	8.2	7.6	5.8	3.8	3.3	2.9	2.4
Cumulative (%)	25.1	45.4	56.9	65.1	72.7	78.4	82.2	85.5	88.4	91.0
Depth	0.1	**0.5**	0.2	0.5	0.0	-0.2	0.1	-0.2	0.3	**0.3**
Temp	0.1	-0.2	0.1	0.2	**-0.9**	0.0	0.1	-0.1	0.1	0.1
Conductivity	**0.9**	0.1	0.3	0.0	0.1	0.0	0.2	0.1	0.1	0.0
TDS	**0.9**	0.1	0.3	0.0	0.1	0.0	0.2	0.1	0.1	0.0
pH	-0.2	**0.6**	0.3	0.3	0.1	**-0.5**	-0.1	0.0	**-0.2**	**0.2**
Eh	0.2	-0.2	0.1	0.2	**0.9**	0.2	0.1	0.0	**0.2**	0.0
DO	0.0	-0.1	**-0.7**	-0.1	**-0.5**	-0.2	**0.3**	0.0	0.2	-0.2
Alk	**0.6**	0.3	**0.6**	0.0	-0.1	-0.1	0.2	-0.2	**0.3**	0.0
Sul	**0.5**	0.2	**0.6**	0.0	-0.3	0.2	0.1	**0.3**	0.0	0.0
Cl	**0.9**	0.1	0.1	0.0	0.2	-0.1	0.1	0.1	-0.2	-0.1
F	0.3	0.2	0.3	**0.5**	**0.4**	-0.2	**0.3**	**-0.3**	-0.1	-0.1
B	**0.5**	-0.3	0.1	-0.1	**0.6**	0.0	-0.3	0.1	0.1	**0.2**
Al	-0.3	**-0.5**	-0.3	0.1	-0.1	-0.2	-0.1	0.0	**0.5**	**0.4**
Ti	0.2	0.1	-0.3	**-0.5**	-0.1	0.1	**0.7**	0.0	0.1	0.1
V	0.2	0.1	**-0.8**	0.3	-0.1	0.1	0.2	0.1	-0.1	0.2
Cr	0.1	-0.2	**-0.9**	0.1	-0.2	-0.2	0.0	0.1	-0.1	-0.2
Mn	-0.1	**-0.5**	0.2	**-0.5**	0.2	**0.3**	-0.1	**0.3**	0.1	**0.3**
Fe	0.0	**-0.5**	-0.4	**-0.4**	0.0	**0.5**	0.0	0.2	**0.2**	0.0
Co	**0.6**	0.0	-0.2	-0.3	0.1	**0.5**	0.3	0.2	-0.1	0.0
Ni	**0.8**	0.1	-0.4	-0.2	-0.1	**0.4**	0.1	0.1	0.1	-0.1
Cu	0.2	-0.2	**-0.7**	**-0.5**	-0.2	**0.3**	-0.1	0.1	0.1	-0.1
Zn	**0.6**	0.0	0.1	-0.2	**-0.4**	0.1	**-0.4**	0.0	**0.3**	0.1
As	0.0	**0.9**	0.1	0.0	0.0	-0.1	0.2	0.0	0.1	-0.1
As^{III}	0.0	0.2	0.3	-0.1	0.1	0.2	**0.2**	**0.7**	**0.3**	0.1
As^{V}	0.0	**0.9**	0.1	0.0	0.0	-0.1	0.1	-0.1	0.1	0.0
DMA^{V}	0.0	**0.9**	0.1	0.1	0.0	0.0	0.2	0.0	0.1	0.0
MA^{V}	**0.4**	**0.5**	0.2	-0.1	0.2	**0.4**	0.1	**0.5**	0.2	0.0
Se	0.1	**0.7**	0.0	0.3	0.0	0.1	0.0	0.0	0.0	**0.5**
Mo	0.1	**0.6**	0.4	**0.4**	-0.1	-0.3	**-0.3**	0.1	0.1	**-0.3**
Ag	0.3	-0.4	-0.1	0.0	0.2	0.1	**0.3**	**0.6**	-0.1	0.0
Cd	**0.4**	-0.2	-0.1	0.0	0.3	0.2	0.0	**0.6**	0.1	**-0.4**
Sn	**0.4**	-0.1	-0.3	-0.1	-0.1	0.0	-0.2	**0.7**	-0.2	0.0
Sb	0.0	**0.8**	-0.1	0.2	0.1	**0.3**	-0.3	0.1	0.0	0.1
Ba	0.2	0.0	**0.9**	0.0	-0.1	-0.2	0.0	0.1	0.0	0.0
W	-0.2	0.0	-0.3	**0.8**	0.0	-0.3	0.0	0.0	0.0	0.1
Pb	0.4	**0.4**	0.0	0.0	0.1	0.2	-0.1	0.1	**0.7**	-0.1
U	0.4	**0.4**	0.0	**0.8**	-0.1	0.2	-0.1	-0.1	0.1	0.0
Mg	**0.5**	0.2	**0.7**	-0.1	-0.2	-0.1	**0.3**	0.0	0.0	0.0
Si	0.3	0.1	0.0	0.2	0.0	0.0	**0.8**	0.1	-0.1	-0.1
Ca	**0.9**	0.0	-0.1	0.2	0.0	0.2	0.0	0.0	0.0	0.0
Na	**0.8**	-0.2	0.0	0.4	0.1	-0.1	0.1	0.2	0.2	-0.1
Sr	**0.4**	0.3	**0.7**	0.2	-0.1	0.0	0.0	0.1	0.0	**-0.3**
K	0.2	-0.1	0.0	-0.1	0.2	**0.9**	0.1	0.1	0.0	0.0

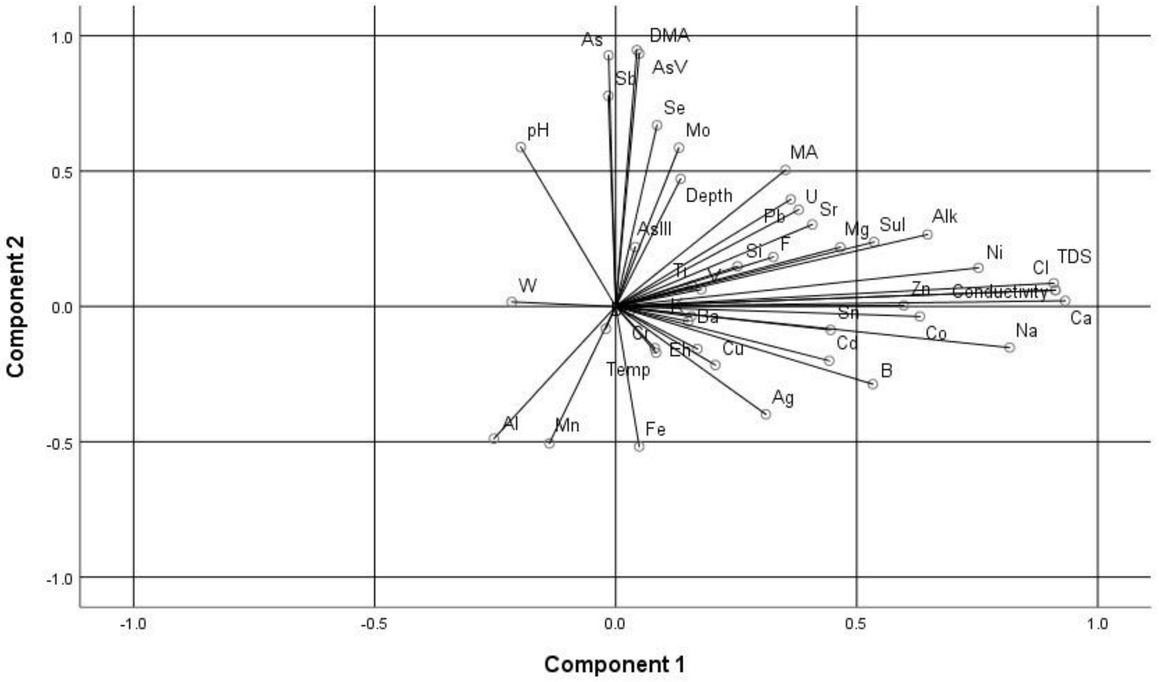

Fig. S6. PCA biplot for 2016 data

References

Cutter G, Andersson P, Codispoti L, Croot P, François R, Lohan M C, Obata H, van der Loeff MR (2010). Sampling and Sample-handling Protocols for GEOTRACES Cruises. GEOTRACES pp. 238 at http://www.geotraces.org/images/stories/documents/intercalibration/Cookbook_v1_2010.pdf [Date accessed 27/09/19].

Environmental Protection Agency (EPA) (2003). Towards setting guideline values for the protection of groundwater in Ireland. Environmental Protection Agency, Wexford, pp. 41.

European Communities (EC) (2010). European Communities Environmental Objective (Groundwater) Regulations, S.I. No. 9 of 2010, pp. 41.

European Communities (EC) (2014). European Union (Drinking Water) Regulations, S.I. No 122 of 2014, pp. 34.
